# Ready-to-Eat Sandwich Microbiota: Diversity, Antibiotic Resistance, and Strategies to Enhance Food Safety

**DOI:** 10.3390/foods15020251

**Published:** 2026-01-10

**Authors:** Ismail M. Al-Bulushi, Zahra S. Al-Kharousi, Mohammed K. Al-Khusaibi, Kamla N. Al-Sarmi, Mohamedsaid Albloushi

**Affiliations:** 1 Department of Food Science & Nutrition, College of Agricultural and Marine Sciences, Sultan Qaboos University, Muscat P.O. Box 34, Oman; isab@squ.edu.om (I.M.A.-B.); mohamedk@squ.edu.om (M.K.A.-K.); 2 Food Safety and Quality Center, Ministry of Agriculture, Fisheries and Water Resources, Muscat P.O. Box 467, Oman; kamillia2008@hotmail.com; 3 Directorate General of Health Affairs, Muscat P.O. Box 314, Oman; 12222@mm.gov.om

**Keywords:** antibiotic resistance, food safety, mitigation, pathogens, quality, sandwiches, virulence genes

## Abstract

Ready-to-eat (RTE) sandwiches are consumed globally due to their convenience, availability, and affordability. Sandwich processing practices and their ingredients expose the sandwiches to various sources of contamination, which can enhance their microbial diversity and introduce certain pathogenic and spoilage bacteria, thereby affecting their safety and quality. Sandwiches may not receive safe cooking temperatures sufficient to destroy food poisoning bacteria, as they are often cooked and served quickly to meet high consumer demand. Improper storage temperatures can enhance microbial growth, and frequent improper handling makes this food a good vehicle for various pathogens such as *Escherichia coli*, *Listeria monocytogenes*, *Salmonella* spp., *Staphylococcus aureus*, and norovirus. Many pathogenic sandwich-associated bacteria, such as *L. monocytogenes*, showed resistance to clinically important antibiotics. Sandwich microbiota have been investigated; however, their diversity, antimicrobial resistance, and importance to sandwich safety and quality have been rarely reviewed. Therefore, this review elucidates the diversity of sandwich microbiota as an impact of ingredients, handling practices, and storage, with emphasis on the importance of this diversity on sandwich safety and quality. It also discusses strategies, control measures, and recommendations to reduce the risk of contamination of sandwiches with pathogenic bacteria or their antibiotic resistance genes, thereby safeguarding public health.

## 1. Introduction

According to the Codex Alimentarius Commission [1], ready-to-eat (RTE) food is defined as any food (including beverages) which is normally consumed in its raw state or any food handled, processed, mixed, cooked, or otherwise prepared into a form in which it is normally consumed without further processing. Ready-to-eat foods are widely consumed worldwide due to their convenience, broad availability, and relatively low prices compared to other conventionally prepared foods. Sandwiches are cooked and served either immediately or stored for subsequent use without any treatment before consumption. Indeed, this processing practice poses some risks that could compromise the safety of the RTE. First, RTE foods are quickly cooked and served to meet consumers’ demands, meaning that RTEs may not receive a recommended safe-cooking temperature that is expected to destroy food poisoning pathogenic bacteria, such as *Salmonella* spp., *Escherichia coli*, *Staphylococcus aureus*, and others, if they are present. Second, RTEs are often not consumed immediately and are probably kept at a temperature above the recommended temperature (below 5 °C), reaching 25–30 °C in some places. Thus, sandwiches may stay at temperatures within the danger zone of 5–63 °C for a time long enough to allow the growth of microbes that survived the cooking temperature either because the heat treatment was not sufficient in killing them or because the contaminant can resist cooking temperature [2].

Various sandwich-associated pathogens can grow significantly under temperature abuse, posing a risk to food safety in sandwich matrices and other RTEs. For instance, the growth of *L. monocytogenes* is significantly influenced by temperature, with higher growth rates observed at elevated temperatures (e.g., 30 °C) compared to lower temperatures (e.g., 4 °C) [3]. Likewise, *S. aureus* can grow at a wide range of temperatures, with growth rates increasing with temperature. For instance, at 30 °C, *S. aureus* can produce staphylococcal enterotoxins in sandwiches, with maximum amounts of 0.15 ng/g after 52 h [4]. The minimum growth temperature for *S. aureus* in dairy products was found to be around 5.7 °C, while storing cheese at temperatures below 0 °C was found to limit maximum population density to approximately 10^2–4^ CFU/g, below the international toxicity threshold [5]. *E. coli* strains, including O157:H7, can survive and grow at various temperatures. However, growth is more pronounced at higher temperatures. *E. coli* O157:H7 showed good growth in ground beef stored at 10 °C [6]. In mixed-ingredient salads stored at 15 °C, *E. coli* O157:H7 populations increased significantly [7]. *E. coli* growth is more pronounced at higher abuse temperatures. In ready-to-eat lettuce stored at temperatures above 16 °C, *E. coli* populations increased up to 1.1 log CFU/g [8].

Storage of RTE sandwiches at high temperatures also leads to the growth of various harmful bacteria, such as *Citrobacter* and *Enterobacter* species, making the sandwiches unfit for consumption [9]. Third, unlike conventionally prepared foods, RTE foods are heavily handled by humans from the first step of ingredient preparation until the serving step. Some researchers studied the effect of food practices of food handlers on the growth of *E. coli* present in tuna sandwiches stored at 4 °C and 30 °C for 6 days. Using good hand hygiene procedures, *E. coli* was only detected when the samples were stored at 30 °C at day 6, while its count remained unchanged from day 0 of storage at 4 °C. In contrast, poor hand hygiene practices lead to the detection of *E. coli* in both temperatures during storage [10].

Ready-to-eat foods include a wide range of foods that differ in their names, main contents, and other ingredients. Sandwiches are considered the typical RTE foods [11] and are processed across the globe with different names, such as shawarma in most Middle Eastern countries or kabab in some Western countries, such as Australia. Sandwiches contain main ingredients such as chicken, meat, tuna, cheese, and eggs, and other secondary ingredients such as bread, salad ingredients, spices, salt, and seasonings. The main ingredients are either barbecued, boiled, or fried and mixed with other secondary ingredients. In cheese sandwiches, the main ingredients are added to the other ingredients without any further treatment. In most developing countries, sandwiches are considered street foods that are prepared outside restaurants and cafes at temperatures that may exceed 40 °C in the summer months under unhygienic conditions and with a lack of food safety knowledge among food handlers. In addition, due to possible unsafe cooking temperature and unhygienic preparation conditions, which provide good chances of cross-contamination from food handlers, utensils, and surfaces, sandwich ingredients are sources and vehicles of pathogens [12,13].

Sandwich main ingredients, such as meat and chicken, and secondary ingredients, such as salad, can significantly contribute to diversifying its microbiota, mainly bacteria, which pose a risk for its safety. For instance, meat and poultry products are the main sources of *Salmonella* spp. [14], whereas salad ingredients such as fresh produce are the main vehicles of sanitary pathogens such as *E. coli* [15]. Moreover, a sandwich is prepared manually by a human who is the main source of *S. aureus,* and these can harbor virulence genes. Other sandwich-associated bacteria, such as *E. coli*, were found to harbor the virulence gene *stx* [16]. As a result, sandwiches are involved in causing many food poisoning outbreaks [17]. Sandwich microbiota has been widely studied in terms of bacterial counts and type; however, the diversity, antibiotic resistance, and food safety aspects were rarely evaluated, highlighted, and reviewed. Therefore, this review aims to address the microbiota of sandwiches, focusing on their diversity, antibiotic resistance, and importance for food safety. It also addresses the health implications for contamination of sandwiches with pathogenic and antibiotic-resistant bacteria, as well as the strategies and control measures to mitigate these risks.

In the culinary context, a sandwich typically refers to a food item consisting of two slices of bread with various fillings in between [18]. In this review, the term sandwich is used broadly to reflect the diversity of ready-to-eat products described in the literature. Across different regions and studies, “sandwiches” encompass foods consisting of bread or a bread-like carrier (e.g., buns, rolls, flatbreads, pita) combined with fillings such as cooked, cured, or processed meats (e.g., deli slices, hot dogs, shawarma, cheeseburgers), poultry, seafood, eggs, vegetables, cheeses, and sauces. They share common characteristics: a multi-component, ready-to-eat assembly with diverse ingredients, minimal further processing before consumption, and potential for microbial complexity arising from multiple contamination sources. The sandwich types included in this review were selected to represent this broad diversity, covering different bread matrices, protein sources, preparation methods, and retail environments reported across global studies. Therefore, the chosen categories capture the major forms of RTE sandwiches encountered in both commercial and household settings.

## 2. Commensal Microorganisms Associated with Sandwiches

Commensal microbes are non-pathogenic microorganisms that naturally inhabit various environments. The background commensal microbiota in sandwiches can vary significantly depending on the type of sandwich, ingredients, and preparation conditions. High aerobic plate counts reaching 6.4 CFU/g were found in various sandwiches, indicating significant microbial presence [19]. The Enterobacteriaceae family includes various commensal bacteria such as *Enterobacter*, *Klebsiella*, and *Citrobacter*, which can be found in food products, including sandwiches. High levels of aerobic plate counts and Enterobacteriaceae indicate poor hygienic conditions during sandwich preparation and storage. For example, aerobic plate count values in sandwiches were found to be satisfactory or acceptable but increased over time during storage [20]. Common genera found in salad include *Enterobacter*, *Acinetobacter*, *Klebsiella*, *Pantoea*, *Achromobacter*, *Microbacterium*, and *Acidovorax* [21]. Lactic acid bacteria (LAB) are frequently found in cooked meat products and are considered beneficial due to their role in fermentation and preservation. They are the dominant group in the microbiota of modified atmosphere packaged sliced cooked meat products, including ham, turkey, and chicken [22].

Cheese harbors a diverse range of commensal bacteria. When cheese is used as an ingredient in sandwiches, these commensal bacteria can be transferred to the sandwich. LAB are the most prevalent microorganisms in dairy products. They include species such as *Lactocaseibacillus casei*, *Lactocaseibacillus paracasei*, and *Lactocaseibacillus rhamnosus*, which are dominant in many ripened cheeses and contribute significantly to flavor development [23]. *Corynebacterium* spp. are commonly found on the surface of smear-ripened cheeses and contribute to the organoleptic properties of the cheese [24]. *Staphylococcus vitulinus* is a non-pathogenic strain that is part of the superficial flora of some cheeses [25]. *Micrococcus* spp. are also found on the surface of various cheeses and contribute to the cheese’s microbial diversity [24]. Bread may harbor various microbes, such as LAB, yeasts, *Acetobacteraceae*, and *Enterococcus*, that can potentially be transferred to sandwiches [26]. Sauces can also introduce a variety of commensal microorganisms. LAB, such as *Lactobacillus* and *Pediococcus*, can be present in soy sauce [27] as well as yeasts [28].

## 3. Spoilage Microorganisms Associated with Sandwiches

Spoilage microorganisms are responsible for the deterioration of food quality, leading to off-odors, off-flavors, and textural changes. Sandwiches contain various ingredients that can be a source of many spoilage microbes. The counts of yeasts and molds reached 4.2 CFU/g in “falafel” sandwiches [19]. Key spoilage microorganisms include *Pseudomonas*, LAB, *Brochothrix thermosphacta*, yeasts, and molds. *Pseudomonas* spp. are aerobic, Gram-negative bacteria known for their spoilage potential in various food products, including meat, fish, and dairy, which are used as ingredients in sandwiches [29,30]. They produce extracellular enzymes such as proteases and lipases, leading to protein and fat degradation [29]. *Pseudomonas fragi*, for instance, is noted for its strong degradation activity in fish, resulting in higher levels of total volatile basic nitrogen and protein oxidation [30]. *B. thermosphacta* is a significant spoilage organism in meat products, particularly under aerobic storage conditions. It produces metabolites like acetoin, contributing to off-odors and spoilage [31]. In co-culture with *Pseudomonas* spp., *B. thermosphacta* can enhance spoilage, leading to higher microbial counts and more pronounced spoilage indicators [32].

While LAB can inhibit other spoilage organisms, they themselves can be spoilage agents in certain conditions, such as in dairy products [33]. LAB thrive in nutrient-rich environments, such as those provided by the ingredients in sandwiches (meat, cheese, vegetables), leading to their proliferation and spoilage activity [34]. Certain LAB strains, such as *Lactobacillus* spp. and *Leuconostoc* spp., can produce slime, leading to a ropy texture in meat products, which can also affect sandwiches containing meat. LAB can grow at refrigeration temperatures, which are typical for sandwich storage. For instance, *Leuconostoc mesenteroides,* which was isolated from cooked meat, could grow well at 4 °C, though at a slow rate [35]. Yeasts and molds are significant contributors to food spoilage, including sandwiches. They produce enzymes that break down lipids and proteins, resulting in off-flavors and odors. Yeasts and molds can tolerate a range of environmental conditions, such as low pH, low water activity, and the presence of preservatives. This extremotolerance allows them to survive and propagate in sandwiches, which often have varied ingredients and storage conditions [36]. For instance, *Hyphopichia burtonii*, *Wickerhamomyces anomalus*, and *Saccharomycopsis fibuligera* were isolated from industrial gluten-free bread, in which they produce dust-type spots of white powdery and filamentous colonies typical of the spoilage produced by chalk yeasts [37].

## 4. Pathogenic Microorganisms Associated with Sandwiches and the Influence of Ingredients, Preparation, Handling, and Packaging

Sandwiches are often prepared and handled at room temperatures ranging from 25 °C to 35 °C. This temperature range enhances the growth of most pathogenic mesophilic bacteria, such as *S. aureus*, *Salmonella* sp., and *E. coli* [18], with humans being a potential source for these bacteria [14,18]. Therefore, many previous studies reported the presence of pathogens and high microbial loads in RTE foods. For instance, poultry products such as chicken and eggs are the main sources of *Salmonella* sp. Meat and meat products are sources of pathogenic *E. coli* and *L. monocytogenes* [18]. Moreover, bread products are a good potential vehicle for *Bacillus cereus* originating from soil [19].

Fresh produce vegetables, which are commonly used in sandwiches, were found to harbor *S. aureus* and various opportunistic pathogens belonging to *Enterobacteriaceae*, such as *E. coli*, *Klebsiella pneumoniae,* and *Enterobacter cloacae* [15], indicating possible contamination from human and animal wastes. Sandwich-associated microbes, which are used to evaluate their safety and quality, can be affected by their ingredients, cooking temperature, hygiene status of handling, and holding temperature before serving. Although aerobic colony count was found to be satisfactory in different sandwiches in Greece [14], Asiegbu et al. [15] reported a serious concern about the safety of sandwiches served in South Africa. Likewise, in Malaysia, Latchumaya et al. [16] found that sandwiches had low microbial quality. Moreover, due to their load of pathogens, Abd-El-Malek et al. [17] raised concerns about the safety of certain types of sandwiches in Egypt. Table 1 shows possible sources and vehicles of microbial contaminants in sandwiches.

Sandwiches such as shawarma are traditionally prepared by slicing meat or chicken, stacking it on a vertical rotisserie with layers of animal fat on the top of the rotisserie for enhancing flavor, slowly roasting until the color of the meat or chicken turns brown, mixing with other ingredients, stuffing in bread, wrapping in paper, and serving. Initially, fat melts and penetrates through cuts, providing a good shelter for microbes from thermal process killing. In fact, bacteria attached to meat surfaces, such as *Salmonella* Typhimurium and *Campylobacter jejuni*, have shown increased heat resistance during cooking, surviving longer than predicted by standard thermal death values. Thus, melting oil could protect bacteria present in meat shawarma by enhancing their thermal resistance and aiding in their recovery post-thermal treatment [47].

**Figure 1:**
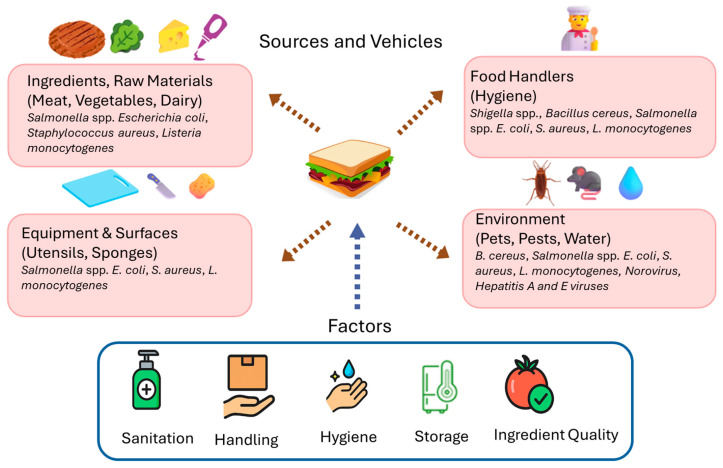
Possible sources, vehicles, and factors affecting microbial contamination of sandwiches [51,52,53,54,55,56].

The temperature of safe cooking is not regularly checked, and the readiness of roasted meat or chicken for serving is indicated by changing the color of the meat or chicken from red to brown. At temperatures lower than 60 °C, myoglobin remains relatively stable, and the meat retains its red or pink color due to the presence of deoxymyoglobin and oxymyoglobin. Cooking meat at higher temperatures leads to the denaturation of myoglobin, resulting in the formation of metmyoglobin. Thus, the color of roasted muscle foods such as meat, chicken, and seafood results from heat-induced changes in myoglobin, such as the oxidation of oxymyoglobin and deoxymyoglobin to metmyoglobin (brown), which does not indicate whether the food reached a safe internal temperature sufficient to kill pathogens. Metmyoglobin formation increases with higher temperatures and prolonged exposure to oxygen [48]. The transition from red/pink to brown is often used as an indicator of doneness and safety. However, this can be misleading. For example, meat cooked in high-oxygen environments can appear brown even at lower temperatures (before reaching the safe internal temperature of 71.1 °C), potentially leading to premature browning and a false sense of safety. Therefore, color change alone is a poor indicator of safety, and a thermometer should be used to ensure proper cooking [49]. Thus, it could be expected that some vegetative and spore pathogens could survive the roasting stage. Moreover, wrapping papers of sandwiches were found to be contaminated with bacteria belonging to the *Bacillaceae*, *Staphylococcus,* and *Pseudomonas* genera [50]. Mayonnaise is a popular seasoning and flavor ingredient in most sandwiches, yet in most sandwich preparation sites, it is made with raw eggs despite regulatory prohibitions. Consequently, mayonnaise was found to cause some food poisoning outbreaks [43]. The possible sources, vehicles, and factors affecting the microbial contamination of sandwiches are indicated in Figure 1.

## 5. Pathogenic Microorganisms in Cook–Serve and Cook–Chill Sandwiches

Based on their ingredients, handling status, and handling temperature, bacteria were the main microbial group targeted in previous microbial research of sandwiches. The main bacterial genera that were investigated qualitatively and quantitatively included *B. cereus*, *S. aureus*, *L. monocytogenes*, *Salmonella* sp., *E. coli*, and Shiga toxin-producing *E. coli*. Therefore, this review focuses on these genera. Moreover, it highlights the importance of sandwich microbiota in food safety and food poisoning outbreaks. Additionally, it elucidates the limitations in the previous studies in the safety and quality evaluation parameters and the need for the determination of more virulence factors in sandwich-associated bacteria. It is also worth noting that although the included studies originate from different countries, several contextual factors can explain variations in microbial contamination. Food service environments differ in hygiene standards, staff training, ingredient sourcing, and the extent of manual preparation. A study from Iran reported good microbial quality in grilled meat and chicken sandwiches possibly due to good preparation practices and effective control and monitoring by food health experts which likely contributes to the satisfactory microbial quality of food products [57]. However, studies from Egypt [58], Jordan [19], and South Korea [59] showed high microbial counts and detection of pathogens in various types of sandwiches, indicating that the current practices may not be sufficient. In addition, a warm and humid climate can support microbial growth in these countries.

### 5.1. Sandwich Prepared Under the Cook–Serve System

Most studies have focused on the microbiota of cooked and immediately served sandwiches, as it is the most common sandwich consumption mode. For instance, to evaluate the effect of Hazard Analysis and Critical Control Point (HACCP) on ensuring the safety of sandwiches, a HACCP-implemented premise sandwich study in Greece did not find the typical pathogens, including *L. monocytogenes*, *Salmonella* sp., and *S. aureus*. The total bacterial and *Enterobacteriaceae* counts were found at an acceptable level [20]. Assessing the microbial quality of different street-vended sandwiches, the cheeseburger was found to contain the highest incidence of *L. monocytogenes*, whereas *Salmonella* sp. was found in hotdog sausages [60]. This indicates the effect of the main ingredients (cheese and sausages) on the predominant pathogen type.

Moreover, to emphasize the effect of the sandwich’s main ingredient, namely poultry and meat products, on the prevalence of its pathogenic microbes, *L. monocytogenes* dominated the *Listeria* sp. in the shawarma prepared from meat and poultry products by 63% and 60%, respectively [58]. Among other pathogens, *Salmonella* Enteritidis, *S.* Typhimurium, *Shigella flexneri*, and *Shigella dysenteriae* were found in liver and minced meat sandwiches [61]. Both *S. aureus* and *B. cereus* were found in 88% of a supermarket’s sandwiches in Taiwan [62]. Moreover, in evaluating the microbial quality of street food in Brazil [63], the prevalences of *E. coli* and *B. cereus* were 18% and 15%, respectively, in hot sandwiches.

In the quantitative aspect, cheese sandwiches were found to contain a higher aerobic mesophilic bacterial count than the fermented sausage sandwiches, which could elucidate the impact of the processing on the sandwiches’ total bacterial load [64]. Moreover, evaluating the microbial quality of kibda (liver) sandwiches, the total bacterial and *Enterobacteriaceae* counts were found in averages of 6 log CFU/g and 4 log CFU/g, respectively [53]. In an RTE microbial surveillance system in the UK conducted for about 2 years, which collected data of 3391 RTE foods, 15.4% of kebab sandwiches were unacceptable based on total bacterial count [65].

### 5.2. Cook–Chill Sandwich

A cook–chill sandwich is cooked and stored at a low temperature until served. The microbiota of this type of sandwich could be affected by storage temperature, besides the previously mentioned factors. In fact, the microbiota of this type of sandwich has not received enough attention compared with cook–serve sandwiches, although some pathogens, such as *L. monocytogenes*, grow normally at chilled temperatures. Moreover, the recommended chill-stored temperature, e.g., 0–3 °C [66], is not followed in many retail stores, especially in developing countries. Thus, storing a cook–chill sandwich in the danger zone, 5–63 °C, which prevails in many developing countries, permits mesophilic pathogens such as *S. aureus* and *Salmonella* sp. to grow. In a small study, total bacterial and *Enterobacteriaceae* counts were found to be high in cook–chill egg, chicken, tuna, and meat sandwiches, and most of the sandwiches were found at the marginal level of acceptability [67]. In another small study, 24 cook–chill sandwiches of different foods, pathogenic *Listeria ivanovii*, *L. monocytogenes*, *S. aureus*, *Yersinia* sp., and *Citrobacter* sp. were identified by Analytical Profile Index (API) strips [11,60]. Both studies have limitations of pathogen coverage and confirmation of the identity of the pathogen by reliable methods, such as genotypic methods. Table 2 summarizes the main pathogens isolated from different types of sandwiches.

Some examples of microbial hazards, growth potential, and risk factors in sandwiches prepared under the cook–serve system as compared to the cook–chill system are given in Table 3.

## 6. Sandwich-Associated Food Poisoning Outbreaks

Most of the sandwich-associated pathogens are part of the common foodborne pathogens that have caused different food poisoning outbreaks globally at high rates. For instance, *L. monocytogenes* caused a historical food poisoning outbreak in South Africa from June 2017 to April 2018, where processed meat products contaminated by *L. monocytogenes* poisoned 937 individuals, resulting in 193 deaths [20]. In 2008, a hospital food poisoning outbreak caused by *B. cereus* in Oman poisoned 58 people. The symptoms included diarrhea and vomiting [80]. *S. aureus* poisoned 24 persons in Italy in 2015, in which three *sea*-positive toxin-producing strains of *S. aureus* were found in the dessert, environment, and cooks, suggesting that the outbreak originated from food handlers [81]. *Salmonella* sp. has dominated food poisoning outbreaks as a causative agent in recent years, and according to the World Health Organization (WHO), a *Salmonella* chocolate outbreak affected 11 countries with 151 cases in 2022. The implicated species was *S. Typhimurium* sequence type 34 [82].

Shiga toxin-producing *E. coli* (STEC) has also been associated with sandwiches. In the United Kingdom, an outbreak of STEC O145:28 was traced back to pre-packed sandwiches and wraps containing lettuce. This outbreak resulted in 288 cases, with 49% of the affected individuals hospitalized and 10% requiring emergency care [83]. Another outbreak in Scotland involved STEC O26:11, where 32 cases were associated with consuming pre-packed sandwiches from a national food chain franchise. The common ingredient in these sandwiches was a mixed salad of Apollo and Iceberg lettuce and spinach leaves [84]. In Japan, about 3000 students were poisoned by *E. coli* serotype O7:H4 carrying the *astA* gene from the consumption of seaweed [85].

Sandwiches with different main ingredients, such as chicken, eggs, and meat, are implicated in a large number of outbreaks. In Taiwan, for instance, 27 people were poisoned by eating online-sold foods. *Salmonella* sp. from egg origin was suspected as the cause of this outbreak [17]. Egg-made mayonnaise is a common dressing item in sandwiches, which could be suspected as a source of food poisoning-causative pathogens. In fact, a sandwich food poisoning outbreak in the UK with 68 cases was caused by *S. typhimurium* DT4 from consumption of sandwiches containing mayonnaise made from eggs [86]. Moreover, at the media level, reports of sandwich-associated food poisoning are widespread. For instance, in 2019, Reuters reported three deaths from hospital sandwiches in the UK. In Hong Kong, Food Safety News reported an outbreak linked to sandwiches that infected 200 people [87]. The Limited Times reported that chicken sandwiches or smoked salmon sandwiches were suspected of poisoning eight individuals in Hong Kong in 2022. Moreover, sandwiches caused a listeriosis outbreak in the USA with a total of 10 people infected and one death [88]. In Spain, a meat-based sandwich was associated with an outbreak of 60 infected people; *E. coli* or *Clostridium perfringens* were possible causes [89].

Outbreaks from sandwiches are commonly caused by a combination of factors such as pathogen contamination, poor food handling and hygiene practices, breaches in cold chain management, environmental contamination, and specific high-risk ingredients. *L. monocytogenes* is frequently found in sandwiches, including hospital settings, leading to severe infections in vulnerable populations [90]. Infected food handlers are a significant factor, with outbreaks often traced back to asymptomatic or symptomatic food handlers who contaminate food during preparation. For example, a restaurant *S.* Enteritidis outbreak was associated with an asymptomatic infected food worker in the United States of America. Moreover, poor hygiene practices, such as not using gloves or improper handwashing, contribute to the spread of pathogens through cross-contamination [91]. Breaches in the cold chain have been linked to the growth of *L. monocytogenes* in sandwiches [90]. Outbreaks have been linked to contamination in the food preparation environment, including equipment and surfaces. An example of that is the outbreak that was caused by *B. cereus* and *C. perfringens* among hospital workers in Alaska in 2021 [92]. Ingredients like egg and poultry are frequently associated with *Salmonella* outbreaks [93], while sandwiches containing salad ingredients, soft cheese, and mayonnaise are more prone to contamination with *Listeria* [90].

## 7. Sandwich Microbial Impact on Its Safety and Quality

The safety and quality of sandwiches have been evaluated by comparison of total bacterial counts, the counts and presence of foodborne pathogens, with the recommended levels in food regulations. Nevertheless, evaluation parameters varied from one study to another. It is also important to note that the cited studies employ different enumeration and detection methods, food matrices, inoculum levels, and storage conditions. To account for this, we interpret each dataset within the context of its respective methodology and focus on identifying consistent patterns rather than making direct numerical comparisons. For instance, Kokkinakis et al. [17] based pre-packed sandwich acceptability on an *E. coli* count of <2 log CFU/g, and an *Enterobacteriaceae* count of 4 log CFU/g was considered borderline. Other pathogens, which were considered safety indicators, included *L. monocytogenes*, *Salmonella* sp., and *S. aureus*; none of them were detected. Khater [20] assessed the quality and safety of liver and kofta sandwiches by counting total aerobic plate, coliform, staphylococci, fungal, proteolytic, and lipolytic microbes. Based on the microbiological guidelines for RTE food and the counts in the previous microbial groups, the contamination level was acceptable in 80% of the liver sandwiches.

In evaluating the bacteriological quality of different sandwiches, Al Harbi et al. [16] considered a count of <5 log CFU/g set by the New South Wales Standards as a criterion of acceptability, and accordingly, none of the sandwiches met this count. Moreover, a combination of qualitative and quantitative microbial parameters such as the counts of aerobic mesophilic bacteria, yeast and molds, *B. cereus*, coagulase-positive staphylococci coliforms and detection of *E. coli*, *Salmonella* sp., and *L. monocytogenes* were used to evaluate the microbiological quality of sandwiches served in hospitals and schools, and according to these parameters, the hygienic conditions of sandwiches were evaluated to be very poor [94]. Contrary to the former study, Bae and Park [53] used *S. aureus* count as a sole criterion to evaluate sandwich acceptability. Besides *S. aureus*, Hanashiro et al. [63] added *B. cereus* as a second bacterium criterion to evaluate the microbial quality of street sandwiches, and accordingly, improving safety was recommended.

On the other hand, certain regional and national standards have been issued to evaluate RTE food safety and quality based on the microbial count and type. For instance, the Gulf Standardization Organization (GSO) Standards request sandwiches with salads to be free from *Salmonella* sp. and *E. coli* O15:H7, but allow *E. coli* at a count of 2 log CFU/g [95]. Food Standards Australia and New Zealand, for example, limit *Enterobacteriaceae*, *B. cereus*, and *S. aureus* counts at <2 log CFU/g [59]. Canadian Standards, however, request *E. coli* count to be <1 log CFU/g, but agree with Food Standards Australia and New Zealand in *B. cereus* and *S. aureus* counts [96].

## 8. Sandwich-Associated Pathogen Virulence Genes

Microbial importance is determined by specific species or strain and their capability to perform specific tasks, such as metabolizing food components to produce products that spoil food or introduce virulence genes to cause foodborne diseases. Certain strains, subtypes or serogroups of the pathogens that have been found in sandwiches in previous studies have been found to harbor specific virulence genes. Among *Bacillus* spp., for instance, *B. cereus* strains harboring virulence genes, such as *nhe*, *hbl*, and *cytK*, were found to be associated with gastrointestinal disorders [97].

Based on the virulence factors and the associated food poisoning outbreaks, *E. coli* was classified into five main groups, namely enterohemorrhagic (EHEC) with the typical Shiga toxin-producing genes; enterotoxigenic (ETEC) with heat-stable genes, *sth*, *stp* and *lt*; enteropathogenic (EPEC) with intestinal microvilli-attaching genes, *eaeA* and *bfpA*; enteroaggregative (EAEC) with an intestinal surface adherence gene, *aggR*; enteroinvasive (EIEC) with a fever and watery diarrheal gene, *ipaH;* and diffuse-adherent (DAEC) with a HEp-2 cell adherence gene, *daaE* [98,99].

*E. coli* Shiga toxin-producing (EHEC) strains are dominating the *E. coli* food poisoning strains. Many standards limited the *E. coli* Shiga toxin-producing strains to *E. coli* O157:H7; nevertheless, with new emerging pathogens, many *E. coli* Shiga toxin-producing strains, which are abbreviated as STEC, such as O26, O45, O103, O111, O121, and O145, are currently causing sporadic food poisoning outbreaks around the globe. Shiga toxin-producing *E. coli* was found to harbor different genes such as *eae*, *aaiC*, *aggR*, *stx2a*, *stx1*, and *stx2*; however, *eae*, *stx1* and *stx2* and *stx2a* genes were found to be involved in a wide spectrum of gastrointestinal disorders ranging from non-bloody diarrhea to hemolytic uremic syndrome. All STEC strains were found to contain *stx* gene and several subtypes of *stx,* such as *stx1a*, *stx1c*, *stx1d*, *stx2a*, *stx2b*, *stx2c*, *stx2d*, *stx2e*, *stx2f,* and *stx2g*, were reported [16,98,100,101,102].

Among the 21 *Listeria* species, *L. monocytogenes* is considered the main pathogenic species, which can cause infection in humans and animals. Moreover, 13 serotypes of *L. monocytogenes* were reported, in which serotypes 4b and 1/2a are widely associated with human listeriosis [103]. The serotype 1/2a was found to produce *InlA*, the gene which is suggested to have a critical role in human listeriosis. *InlA* was found in 96% and 65% of the clinical and food strains, respectively [104]. Along with *InlA,* which facilitates *L. monocytogenes* attachment to the host cell, *prfA* and *hly* genes were found to facilitate the intracellular pathogenesis potential of *L. monocytogenes* [103]. Staphylococcal food poisoning was widely found to be caused by a wide variety of *se* genes ranging from *sea* to *see* and *seh* worldwide; however, the *sea* gene was found to be involved in the most staphylococcal food poisoning outbreaks [81,105,106,107]. Within these genes, *sea* and *see* were found mostly in food and clinical samples [105]. Almost all *Salmonella* species are pathogenic and have been associated with the most frequent salmonellosis from different sources around the globe. Table 4 summarizes the pathogens’ main virulence genes.

## 9. Antibiotic Resistance in Sandwich-Associated Bacteria

Antibiotics are chemical substances produced by various microbes. They are utilized to treat infections in humans and animals or as a prophylaxis measure to prevent them [109,110]. Unwise use of antibiotics, such as utilizing sub-therapeutic concentrations to enhance animals’ growth, may contribute to the development of antibiotic resistance in animal-associated microbes and to the build-up of antibiotic residues in the edible animal tissues [100]. “Resistance” refers to the microbial inherited capability to grow at increased concentrations of antibiotics, irrespective of the period of the treatment. It is usually determined by evaluating the minimum inhibitory concentration (MIC), which is the lowest concentration required to inhibit the visible growth of a microbe for a particular antibiotic. The term “resistome” is used to refer to the collection of genes involved in various mechanisms of antibiotic resistance [111]. Infections caused by antibiotic-resistant bacteria (ARB) can increase morbidity and mortality rates because of the possibility of treatment failure. These infections are also costly [74]. The increased bacterial antibiotic resistance burden is attributed to the tremendous use of antibiotics in agriculture, animals, and humans [112].

Microbial antibiotic resistance is well documented in different pathogenic microbes isolated from different foods. This resistance has been attributed to many factors, such as climate change, which has received special attention. In fact, a clear association between antibiotic resistance increase and climate change was found and emphasized in many studies [113,114,115,116]. For instance, an increase of 1 °C in ambient temperature increased certain pathogens’ antibiotic resistance, such as *Pseudomonas aeruginosa*, by 1.06-fold [113]. This association was attributed to the impact of temperature increase due to climate change on increasing microbial growth and adaptation, as well as pathogenic gene exchange and antibiotic resistance among microbial populations [113,117]. Antibiotic resistance in sandwich bacteria was widely investigated worldwide [11,118,119,120,121]. The pathogens that showed the most antibiotic resistance included *S. aureus*, *L. monocytogenes,* and *E. coli* [11,118,119]. Certain antibiotics which showed resistance in previous studies, such as amoxicillin, metronidazole, and tetracycline, are clinically important in Oman and other countries [122].

### 9.1. Prevalence of Antibiotic Resistance in Ready-to-Eat Foods and Sandwich-Associated Bacteria

The prevalence of ARB associated with RTE sandwiches is a significant concern, as these foods can serve as vectors for resistant pathogens. Nevertheless, there is limited data in the literature concerning antibiotic resistance in sandwiches as compared to other RTE foods and sandwich ingredients. This highlights the need for stringent food safety measures and routine surveillance, including larger geographical locations and large-scale studies, to mitigate the risks associated with ARB in sandwiches. A study evaluating bacterial load in RTE sandwiches from vending machines and supermarkets in Modena, Italy, found that 50% of the 54 bacterial isolates were pathogenic. These included *L. ivanovii*, *L. monocytogenes*, *S. aureus*, *Yersinia* spp., *Citrobacter* spp., and *Enterococcus* spp. Two *Enterococcus faecium* isolates exhibited resistance to vancomycin, and one isolate showed resistance to both ampicillin and erythromycin. One *S. aureus* isolate had resistance to erythromycin (2 µg/mL) and oxacillin. Additionally, five *Listeria* isolates demonstrated resistance to erythromycin. *Aeromonas hydrophila* was resistant to imipenem, and *Citrobacter* showed resistance to amikacin [52]. These findings highlight the potential health risks associated with consuming contaminated sandwiches.

Studies from Iran [123] highlight the role of both ready-to-eat (RTE) foods and raw sandwich ingredients as important reservoirs of antibiotic-resistant foodborne pathogens. *S. aureus* has been detected in a variety of RTE products in Tehran, with notable levels of contamination and widespread resistance to commonly used antibiotics, including penicillin, tetracycline, gentamicin, and several others. These findings indicate that consumers of RTE foods may be exposed to multidrug-resistant *S. aureus* through improperly handled or prepared items. Complementary investigations [124] of raw kebab and hamburger meat that are frequently used in sandwich preparation also revealed high contamination rates with *E. coli*, *Salmonella* spp., *L. monocytogenes*, and *S. aureus*. Many of these isolates carried clinically important resistance determinants such as *bla*_SHV_, *bla*_TEM_, and *mecA*, highlighting the circulation of β-lactamase-producing and methicillin-resistant strains within the meat supply. Together, these studies demonstrate that both raw meat components and RTE products commonly used in sandwiches can serve as significant vehicles for the transmission of antibiotic-resistant bacteria, underscoring the need for improved hygiene, handling, and monitoring practices across the sandwich production chain.

Evidence from Egypt [72] further demonstrates the substantial public health risk posed by antibiotic-resistant *S. aureus* in RTE sandwiches. In an assessment of beef burger and hot dog sandwiches, coagulase-positive *S. aureus* was detected in the majority of samples, with a large proportion classified as multidrug-resistant. Notably, the presence of methicillin-resistant *S. aureus* (MRSA) and even vancomycin-resistant *S. aureus* (VRSA) highlights the circulation of highly resistant strains in foods that are consumed without further cooking. The isolates exhibited extensive resistance to multiple antibiotic classes, suggesting significant antimicrobial selection pressure within the food chain. The detection of MDR, MRSA, and VRSA in popular sandwich items underscores the potential for RTE foods to act as vehicles for clinically important resistant pathogens and highlights the urgent need for stricter hygiene controls, improved handling practices, and surveillance measures to protect consumers

Several studies report the presence of Extended-Spectrum β-Lactamases (ESBLs)- and AmpC β-Lactamases (AmpC)-producing Enterobacteriaceae in ready-to-eat (RTE) sandwiches, indicating that these foods can act as vehicles for antibiotic-resistant pathogens. For example, in Algeria [44], *E. coli*, *K. pneumoniae*, and *K. oxytoca* isolates recovered from street-vended sandwiches frequently carried CTX-M, SHV, and AmpC β-lactamase genes, suggesting circulation of multidrug-resistant clones within the food chain. The detection of identical ESBL/AmpC-producing strains in sandwiches from different city locations also highlights widespread contamination likely originating from shared ingredients, inadequate hygiene during preparation, and cross-contamination during handling or packaging. Overall, these findings underscore the need for improved control measures and monitoring of RTE sandwiches to reduce consumer exposure to resistant pathogens. A summary of antibiotic-resistant foodborne pathogens recovered from sandwiches is provided in Table 5.

### 9.2. Mechanism of Antibiotic Resistance in Foodborne Bacteria

The correlation between the increased use of antibiotics and the emergence of ARB is widely acknowledged [112]; however, antibiotics, ARGs, and the mechanisms responsible for the transmission of ARGs between bacteria have been present for millions of years before humans started to use antibiotics [126]. In their natural niches, bacterial populations are usually heterogeneous in their susceptibility to antimicrobial agents [127]; however, when they are challenged with antibiotics, resistant cells outcompete the susceptible counterparts in a ‘Darwinian’ fashion [110]. The function and the origin of antibiotics and ARGs cannot be certified. Their existence in the natural environments, including those that have not been visited previously by humans, can be explained by two main reasons: (1) Some bacteria may produce antibiotics to compete with other microorganisms for the limited nutrients. These antibiotic producers, at the same time, develop mechanisms to counteract the effect of the antibiotics they synthesize, rendering them resistant to antibiotics [128]. (2) Antibiotics may be involved in or perform significant functions for the bacterial cells that produce them, such as signaling [129] and biofilm formation [126]. Nevertheless, other bacteria produce other compounds that have functions involved in cell signal transduction, homeostasis, and metabolism, but these molecules can also resist high concentrations of antibiotics. The existence of ARGs in extreme environments that are not known to be polluted by humans, such as deep Greenland ice core and clean Antarctic water, is an example [129]. Bacterial resistance to antibiotics can be an intrinsic criterion [130], or it can be developed through gene acquisition, accumulation of mutations, or both, utilizing various mechanisms [98,112,126].

#### 9.2.1. Intrinsic Bacterial Resistance to Antibiotics

Intrinsic bacterial resistance occurs when bacteria resist a particular antibiotic due to structural or functional characteristics of these bacteria [93]. Intrinsic resistance is not related to the use of antibiotics; instead, it is a natural, complicated process exhibited in all bacteria, especially environmental bacteria, including food bacteria. The outer membrane, which acts as a permeability barrier in Gram-negative bacteria, and efflux pumps are the most common intrinsic antibiotic resistance mechanisms [131]. Efflux pumps have the potential to pump antibiotics or other antimicrobial substances, such as disinfectants, outside the cell. They utilize adenosine triphosphate (ATP) or proton-motive force (PMF) to obtain energy to complete this process [20]. Efflux pumps are grouped into two main families: (1) the major facilitator superfamily (MFS) and (2) the small multidrug resistance (SMR) family [132]. Many genes are involved in the intrinsic resistance mechanism [130,131], which can be targeted by manufacturing antibiotics to inactivate specific pathogens [93]. Various serotypes of *E. coli* were found in RTE sandwiches, with intrinsic resistance to antibiotics like erythromycin. Being a Gram-negative bacterium, *E. coli* has an outer membrane that prevents macrolides like erythromycin from entering the cell. Moreover, efflux pumps also contribute to reduced susceptibility [121]. *Staphylococcus* spp. and *Enterococcus* spp., which are widely isolated from sandwiches, use efflux pumps to expel antibiotics, contributing to intrinsic resistance. *Acinetobacter* spp., known for their intrinsic resistance to multiple antibiotics, were also isolated from RTE sandwiches. These bacteria are particularly problematic in healthcare settings due to their resistance profiles. Interestingly, intrinsic genes coding for antibiotic resistance could be the origin of acquired resistance, particularly in the genus *Acinetobacter* [133]. This can happen through horizontal gene transfer (HGT) when intrinsic resistance genes are mobilized and transferred to other bacteria via plasmids, transposons, and integrons or through incorporation of the intrinsic resistance genes into mobile genetic elements, which facilitate their spread among different bacterial populations [134].

#### 9.2.2. Acquired Bacterial Antibiotic Resistance

Acquired bacterial antibiotic resistance can occur via three main mechanisms: (1) modification in the antibiotic target site by post-translational modification or mutations, (2) decreasing the intracellular level of antibiotic by utilizing efflux pumps or reducing permeability, or (3) antibiotic inactivation by hydrolysis or modification [130]. Natural selection and evolution largely depend on mutations that occur in bacterial DNA. Bacteria are haploid for their genes with short generation times, which leads to the accumulation of mutations, thus increasing the potential of the emergence of antibiotic-resistant phenotypes [135]. The rate of gene mutation is affected by factors including environment and population dynamics, microbial genetic makeup, and cell physiology. For a full resistance to occur, a mutation should occur in multiple genes, because a single antibiotic has genetic redundancy in its target sites. For instance, the antibiotic fluoroquinolone targets the enzymes topoisomerase II and IV that are important for supercoiling of bacterial DNA and are encoded by the genes *parA*, *parC*, *gyrA*, and *gyrB*. Mutations should occur in at least two of these genes or all of them for the development of a bacterial phenotype that is fully resistant to fluoroquinolone [136].

A bacterial ‘hypermutable’ phenotype may arise when bacteria have defects in their DNA mismatch repair system, leading to potential accumulation of significant mutations [135]. Mutational resistance can develop to some antibiotics, such as streptomycin, fusidic acid, and rifampicin, when they are used during a treatment course against certain bacteria, and thus the combination of these antibiotics with these bacteria should be avoided [137]. Studies showed that exposure to osmotic pressure and freezing stress significantly affects the antibiotic resistance of *S. enteritidis* and *S. typhimurium*. Prolonged freezing (96 h) increased antibiotic resistance, while shorter freezing periods (24 h) decreased it [138]. This suggests mutation-based antibiotic resistance can arise in food-associated bacteria such as *Salmonella* and *Campylobacter*, especially under environmental stresses (e.g., salt, low pH, heat, and preservatives). However, detecting natural mutation events in sandwich-associated bacteria and identifying the specific genes involved remain active research areas that require further investigation.

#### 9.2.3. Antibiotic Resistance Development by Horizontal Gene Transfer

Lateral or HGT is the process of transferring antibiotic resistance between different bacterial cells. Transformation, transduction, and conjugation are the three principal mechanisms for HGT. Transformation occurs when naked DNA that contains resistance determinants is released to the environment and taken up by a recipient bacterial cell [126], which should enter a ‘competence’ state. Then, particular recognition sequences are recognized in the DNA for successful transformation [136]. DNA released from dead cells to the environment was observed to persist and to be protected by soil particles from DNase [95]. Transduction happens when a bacteriophage is involved in transferring antibiotic resistance genes from one bacterial cell to another [126,136]. Lysogeny is the process of integrating the genes into the chromosome of the recipient bacterial cell [126]. Bacteriophages can mediate the transfer of antibiotic resistance genes among Enterobacteriaceae, including non-typhoidal *Salmonella* and Shiga toxin-producing *E. coli*. This mechanism significantly contributes to the spread of resistance genes in foodborne pathogens [139]. Conjugation involves the transfer of antibiotic resistance genes that are located on a plasmid or conjugative transposon that occurs when these elements are moved from a donor cell to a recipient one via a mating bridge [126,136]. In fact, the food environment may facilitate HGT involving various species. For example, a transposase gene was transferred from *Enterococcus* to *Streptococcus thermophilus*, and a plasmid was transferred from *S. thermophilus* to *E. faecium* [140]. A fourth process of HGT has been mentioned, which occurs through DNA-containing membrane vesicles that are released from the surface of bacterial cells and acquired by other bacteria [141]. Outer membrane vesicles (OMVs) from *Campylobacter coli* were demonstrated to transfer both plasmid-encoded and chromosomally encoded antibiotic resistance genes to *Campylobacter jejuni*. This transfer is independent of natural transformation and involves direct fusion between OMVs and recipient bacterial membranes. *Campylobacter* is an important foodborne pathogen whose resistance to antibiotics poses a serious threat to public health [142].

Transposons are important genetic elements in the context of HGT. They are special genetic elements that have the capability of excising themselves from their genetic loci into new loci either in the same bacterial cell or in other bacteria, even those of different taxa. They can be transferred by the three described basic mechanisms of HGT and are known to play a significant role in the development of antibiotic resistance. This is because they have special gene sequences called “integrons”, which are particularly important in the dissemination of antibiotic resistance genes between bacteria [136]. Resident plasmids can be mobilized by conjugative transposons [143]. Additionally, circular gene cassettes can be moved among bacteria. Multidrug resistance can occur because of the integration of multiple gene cassettes [136] that are transferred together [11,129]. A superintegron may have more than 100 antibiotic resistance genes [11].

**Figure 2:**
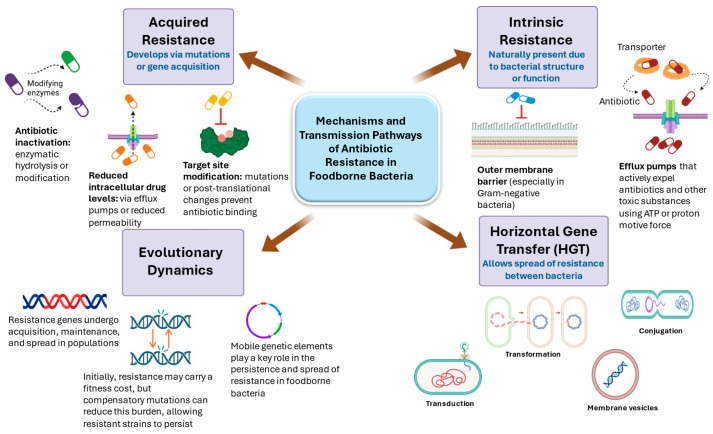
Summary of the mechanisms and transmission pathways of antibiotic resistance in foodborne bacteria.

The three main steps that determine the fate of an antibiotic-resistant gene in an environment are the acquisition, maintenance, and spread of the gene [118]. Initially, the biological cost of having an antibiotic resistance gene is high [144], but some mechanisms of resistance have a low fitness cost and thus the wild antibiotic-sensitive bacterial types will not outcompete the antibiotic-resistant bacteria [129]. This is because, to lessen the negative effects of the resistance mutations, compensatory mutations occur in antibiotic-resistant mutants [135]. The presence of plasmids and transposons as mobile transfer elements carrying antibiotic resistance genes was demonstrated in many foodborne bacteria [110]. Figure 2 summarizes the mechanisms and transmission pathways of antibiotic resistance in foodborne bacteria.

ARB have been isolated from a wide range of habitats, including humans, animals, plants, natural ecosystems, and food environments [145]. This broad distribution underscores the role of commensal, spoilage, and pathogenic bacteria in disseminating ARGs and highlights the food chain as an important conduit linking environmental and human reservoirs of resistance [146]. The multilayered matrices of ready-to-eat foods such as sandwiches, which contain diverse ingredients supplying nutrients, moisture, and structurally complex surfaces, can create favorable microenvironments for HGT among resident microbial communities. Evidence from fresh-produce studies illustrates this risk. Chopped lettuce has been shown to support HGT of ESBL genes, such as *bla_SHV-18_*, between *K. pneumoniae* strains, with transfer frequencies increasing under mild temperature abuse (15–24 °C). Notably, gene transfer on lettuce occurred at even higher frequencies than in liquid media, indicating that fresh produce can constitute an effective environmental surface for the propagation of antibiotic resistance [147].

Chicken meat has been shown to carry phages capable of transferring ARGs. Approximately 24.7% of phages isolated from chicken meat were able to transduce resistance to antibiotics such as kanamycin, chloramphenicol, tetracycline, and ampicillin into *E. coli* [148]. This suggests that phage-mediated HGT is a significant pathway for spreading ARGs in chicken meat. In dairy products like cheese, HGT can occur through conjugative plasmids. For instance, in Minas Frescal cheese, integrons carrying ARGs for β-lactams, tetracyclines, quinolones, and sulfonamides were detected, indicating potential HGT events [149]. These integrons facilitate the transfer of resistance genes among bacteria in the cheese matrix. In pickled vegetables, metagenomic sequencing has identified the presence of ARGs and mobile genetic elements that facilitate HGT. For example, *Levilactobacillus brevis* in pickled vegetables carried MDR genes and transposable elements, indicating active HGT processes [150]. Within sandwiches, commensal or spoilage bacteria may thus serve as intermediate hosts facilitating ARG exchange with pathogenic species, which can then cause infections or exchange ARG with other pathogenic or non-pathogenic microbes. A well-known example is the global dissemination of the *bla*CTX-M-15 gene, encoding a dominant ESBL enzyme. Originally located on the chromosome of environmental *Kluyvera* spp., this gene mobilized onto plasmids and subsequently spread to diverse clinical pathogens worldwide, emerging as the most significant acquired resistance mechanism to third-generation cephalosporins since its first identification in the mid-1990s [151].

Many studies have demonstrated the presence of phenotypic and genotypic antibiotic resistance among bacteria isolated from RTE sandwiches [145]. Beyond their presence in the final product, these resistance determinants may persist and exert effects after ingestion. Certain strains can act as commensals, opportunistic pathogens, or primary pathogens that colonize the human gastrointestinal tract, where infections may occur even long after exposure to contaminated food [152]. Importantly, ingested ARB and their associated genes can contribute to the intestinal resistome, particularly when resistance determinants persist and integrate into the gut microbial community [153]. These bacteria or free DNA may also serve as vehicles for the horizontal transfer of ARGs within the gut [154] or among microbial populations present on sandwich ingredients such as fresh produce [147]. Because RTE sandwiches typically undergo minimal or no heat treatment, they are more likely to introduce viable ARB and intact ARGs into the gastrointestinal tract compared to foods exposed to higher thermal processing, where DNA degradation is more extensive [152]. Consequently, RTE foods can facilitate the spread of ARB or ARGs beyond the point of consumption, with potential implications for public health, including reduced treatment efficacy, higher healthcare costs, and increased morbidity and mortality associated with resistant infections [155].

### 9.3. Public Health Implications of Antibiotic-Resistant Bacteria in Sandwiches

Sandwiches can be a silent vehicle for spreading MDR bacteria. Antibiotic resistance in RTE foods, including sandwiches, poses significant public health risks. The presence of ARB in these foods can lead to severe health consequences for consumers, particularly those who are immunocompromised or elderly. Consumption of RTE foods contaminated with ARB can lead to foodborne illnesses that are difficult to treat due to resistance. This can result in longer hospital stays, higher medical costs, and increased mortality rates [156]. Moreover, antibiotic resistance can enhance the pathogenicity of bacteria, increasing the severity of infections. For example, resistant strains of *E. coli* and *S. aureus* can produce toxins that exacerbate food poisoning symptoms [71,125]. A study examined 140 RTE sandwiches (burger, hawawshi, kofta, liver, luncheon, shawarma, and sausage) in Egypt for enterotoxin producers and MDRSA (multidrug-resistant *S. aureus*). One or more staphylococcal enterotoxin genes were detected in 72.7% of *S. aureus*, and the *mecA* gene was detected in 81.8% of coagulase-positive *S. aureus*. These bacteria also showed resistance to various antibiotics such as kanamycin (100%), penicillin and neomycin (92%), oxacillin and erythromycin (84%), and ampicillin and nalidixic acid (68%) [71].

Likewise, in a study conducted to investigate the presence of MDRSA in 60 RTE shawarma sandwiches in Malaysia, 60% of the samples harbored *S. aureus,* with 80.6% of them being resistant to at least one antibiotic. Resistance was demonstrated for ampicillin (69.4%), penicillin (69.4%), ciprofloxacin (47.2%), tetracycline (33.3%), kanamycin (22.2%), trimethoprim (5.6%), trimethoprim–sulfamethoxazole (2.8%), gentamicin (2.8%), and cephalothin (2.8%). Moreover, 33.3% of the isolates were MDR and were biofilm producers [52]. These results show the potential of ARB present in RTE foods to harbor various virulence factors and their genes, complicating infections and treatment outcomes, as well as making their eradication more difficult. Therefore, vulnerable populations, such as the elderly and immunocompromised individuals, are at higher risk of severe outcomes from infections caused by resistant bacteria [11]. Moreover, the consumption of contaminated RTE foods can facilitate the spread of antibiotic resistance genes among human populations, exacerbating the public health crisis [157]. RTE foods contaminated with resistant pathogens have been linked to outbreaks of foodborne illnesses, highlighting the need for stringent food safety measures [83]. Various foodborne pathogens, such as *L. monocytogenes* [158], *E. coli* O157:H7, and *S. aureus* [159] capable of causing outbreaks, have been demonstrated to be MDR. Thirty-nine *V. parahaemolyticus* isolates were recovered from 511 RTE Chinese foods from 24 cities, and the isolates exhibited resistance to ampicillin (51.3%), cefazolin (51.3%), and streptomycin (89.7%).

### 9.4. Strategies, Control Measures, and Recommendations to Reduce Risk of Antibiotic Resistance in Sandwiches and Other RTE Foods

To mitigate the risk of contamination of sandwiches and other RTE foods with pathogenic bacteria, particularly those exhibiting antibiotic resistance traits or their genes, various strategies and frameworks can be targeted. First, it is crucial to strengthen surveillance systems and regulatory frameworks to monitor and control antibiotic resistance in the food supply chain in every country [5,157,160]. Antibiotic resistance is a global challenge, and the development of resistance in one country can spread to other countries. In alignment with the efforts of the international organizations (the World Health Organization, the World Organization for Animal Health, the Food and Agriculture Organization of the United Nations, and the Codex Alimentarius Commission) in establishing standards for resistance surveillance programs, it will be important to establish integrated surveillance programs that harmonize laboratory testing methodologies and antimicrobial-use reporting across different sectors, including human health, animal health, and food production [161,162].

To ensure consistency and comparability of data across nations, it is advisable to utilize existing frameworks like the Global Antimicrobial Resistance and Use Surveillance System (GLASS) [163,164]. It is necessary to implement a comprehensive monitoring systems that cover the entire farm-to-fork continuum, including sampling from humans, animals, and food products [162] as well as to develop real-time surveillance and clinical decision-support systems, such as HAITooL, to monitor antibiotic usage and resistance rates, facilitating early identification of outbreaks and supporting proper antibiotic prescription [165]. Moreover, the surveillance program can consider wastewater surveillance as a complementary tool for tracking antibiotic-resistant genes and bacteria, providing early warnings of emerging threats [166].

Second, implementing responsible and safe food production practices, including the prudent use of antibiotics in agriculture, can help mitigate the spread of resistance [157,167]. In this case, developing regulatory frameworks can be powerful to enforce stringent policy interventions governing antibiotic usage in food production, including registration management policies, usage monitoring systems, and integrated surveillance programs [160]. Policy makers can also learn from successful stewardship strategies in European nations, such as banning antibiotics for disease prevention, benchmarking antibiotic utilization, and setting national reduction targets [168]. Tackling the antibiotic resistance problem also needs international collaboration and coordinated action to address antibiotic resistance, leveraging the One Health approach to integrate efforts across human, animal, and environmental health sectors [169]. To ensure that regulatory measures are contextually appropriate and effective, it is important to address economic dependencies and cultural understandings of risk that drive global antibiotic consumption and resistance [170].

**Figure 3:**
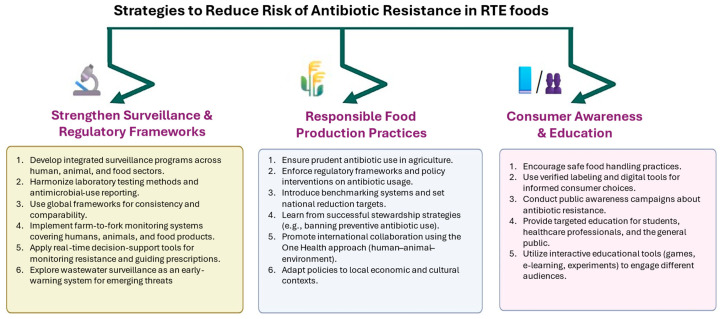
Summary of the strategies to reduce the risk of antibiotic resistance in sandwiches and other RTE foods.

Third, raising awareness about the risks associated with antibiotic-resistant bacteria in RTE foods and promoting safe food handling practices among consumers is essential [157]. This can be achieved by enhancing consumer awareness and responsibility through verified labeling and digital innovations that support informed choices regarding antibiotic use in food products [168] and conducting public educational campaigns to improve knowledge and behaviors related to antibiotic use and resistance, leveraging interactive and engaging methods [171]. Moreover, targeted education programs can be implemented for various stakeholders, including students, healthcare professionals, and the general public, to improve understanding and practices related to antibiotic use. Diverse educational tools, such as board games, e-learning, and environmental experiments, to engage different audiences and promote responsible antibiotic behavior, can be used [169]. By integrating these strategies, surveillance systems and regulatory frameworks can be significantly strengthened to monitor and control antibiotic resistance in the food supply chain, ultimately contributing to global public health and food safety. The strategies to reduce the risk of antibiotic resistance in sandwiches and other RTE foods are summarized in Figure 3.

It is also important to note that effective control of pathogenic and ARB in RTE foods requires targeted interventions. Several validated strategies have demonstrated success in reducing ARB and ARGs in RTE food systems. Hurdle technologies that combine pH reduction, lowering water activity, and applying organic acids have been shown to suppress *L. monocytogenes* in RTE meats and sandwiches [172]. High-pressure processing (HPP) is widely recognized for its ability to inactivate pathogens while preserving sensory quality, with documented reductions of up to 5-log CFU for various foodborne pathogens [173]. Antimicrobial packaging incorporating nisin or plant-derived compounds has effectively reduced *Listeria* growth on RTE cheeses and fresh produce during storage [174]. In ingredient preparation, interventions such as peracetic acid washes, chlorine dioxide, and UV-C treatment significantly reduce microbial loads on fresh produce commonly used in sandwiches [175,176]. Improved sanitation protocols, cross-contamination controls, and temperature management along the preparation chain further minimize risks [177].

Bacteriophage therapy can be effective against multiple serotypes of ARB in RTE foods like sandwiches. Several commercial phage preparations (e.g., those targeting *L. monocytogenes* and *Salmonella*) have been approved for application on RTE meats, fish, fresh produce, and dairy products. These phages can be applied as surface sprays or dips and have demonstrated significant log-reductions in pathogen levels without affecting sensory quality. Importantly, phage therapy offers high specificity, minimizing disruption to beneficial microbiota and reducing selective pressure associated with chemical sanitizers [178]. Incorporating these techniques into sandwich preparation systems along the food chain can reduce the risk of consuming sandwiches containing ARB or their genes, thereby protecting consumer health. In addition, global surveillance systems such as GLASS can be operationalized by integrating AMR monitoring into raw-material testing, facility-level environmental sampling, and post-processing verification.

## 10. Previous Studies’ Limitations

Despite its widespread consumption as a typical RTE food in many countries around the world, there is still a lack of comprehensive studies regarding the microbiota of sandwiches that include commensal, opportunistic, and pathogenic microbes in many countries, such as Oman. Most investigations focused on pathogenic bacteria that are mostly involved in causing outbreaks. In general, previous studies investigating the microbiota of sandwiches have faced several limitations, which can be categorized into methodological, environmental, and analytical challenges. Regarding methodological limitations, many studies have limited sample sizes and often focus on specific types of sandwiches or ingredients, which may not represent the full diversity of sandwich types and their microbiota. For instance, studies often examine a narrow range of sandwich types, such as falafel or pre-packed sandwiches, without considering the wide variety of sandwiches consumed globally [19,20]. The conditions under which samples are collected and stored can significantly impact the microbial load. For example, the storage location and temperature can affect the bread’s structure and moisture content, which in turn influences microbial growth [179]. Additionally, the time between sample collection and analysis can vary between studies, leading to changes in the microbial community [19]. The techniques used to detect and enumerate microorganisms can vary, leading to inconsistencies in results. Different studies use various media and methods for microbial enumeration, which can affect the detection of specific microorganisms [19,20]. For example, the use of different agar media types for counting mesophilic aerobes, coliforms, and *S. aureus* can yield different results and thus interpretations [19].

Environmental limitations, such as the hygienic conditions of the environment where sandwiches are prepared and sold, can greatly influence the microbial load. Studies have shown that sandwiches from street vendors or certain restaurants often have higher microbial counts due to poor hygienic practices [58]. This variability makes it challenging to generalize findings across different settings. Moreover, there is the issue of ingredient variability. The type and quality of ingredients used in sandwiches can introduce variability in microbial communities. Ingredients like hummus and tahini salad have been shown to contribute significantly to the microbial load in falafel sandwiches [19]. Similarly, the presence of additional ingredients like tomatoes in pre-packed sandwiches can increase microbial counts [20].

Analytical limitations may be due to microbial community complexity. The complexity of microbial communities in sandwiches poses a challenge for comprehensive analysis. Traditional culturing methods may not capture the full diversity of microorganisms present, leading to an incomplete understanding of the microbiota. Advanced techniques like metagenomics can provide a more detailed picture, but are not always used [180]. Another analytical limitation is pathogen detection. While some studies focus on specific pathogens like *L. monocytogenes* and *Salmonella* spp., the absence of these pathogens in samples does not necessarily indicate overall microbial safety. The presence of other harmful microorganisms, such as *S. aureus* and *B. cereus*, can still pose significant health risks [20].

## 11. Conclusions

Sandwich ingredients and their preparation practices were found to diversify their microbiota with the prevalence of pathogens such as *S. aureus*, *Salmonella* sp., *E. coli,* and *L. monocytogenes*. Moreover, microbiota diversity was found to cause many food poisoning outbreaks globally. Many sandwich-associated pathogens, such as *B. cereus*, *S. aureus*, *L. monocytogenes,* and STEC *E. coli*, were found to possess virulence factors such as *Nhe*, *sea, InlA* and *eaeA*. Most sandwich-isolated pathogens, which showed antibiotic resistance, included *S. aureus*, *L. monocytogenes,* and *E. coli*. Sandwich-associated pathogens showed significant resistance to commonly used antibiotics, such as penicillin, tetracycline, gentamicin, erythromycin, trimethoprim–sulfamethoxazole, and ciprofloxacin. To minimize the presence of pathogens with antibiotic resistance in sandwiches, the current review proposed certain strategies, such as strengthening surveillance systems, implementing responsible, safe food production practices, raising consumer awareness about the risks associated with ARB in RTE foods, including sandwiches, and promoting safe food handling practices among consumers.

## Figures and Tables

**Table 1:** Possible sources and vehicles of microbial contaminants in sandwiches.

Stage/Source	Food/Surface	Role	Most Possible Contaminant	References
Ingredients	Meat	Source	*Listeria monocytogenes*, *E. coli*, Shiga toxin-producing *E. coli* (STEC)	[18,19]
	Poultry products	Source	*Salmonella* sp.	[38]
	Tuna	Source	*Clostridium botulinum*	[39]
	Salad (tomato, cucumber, cabbage, lettuce)	Vehicle	Noroviruses, hepatitis A, *E. coli*, Shiga toxin-producing *E. coli* (STEC), *Salmonella* sp.	[40,41]
	Bread	Source	*Bacillus cereus*	[42]
	Mayonnaise	Vehicle	*Salmonella* sp.	[43]
	Spices	Vehicle	*B. cereus*	[44]
Preparation	Cutting board and knife	Vehicle	Serves as a vehicle for all the above microbes	[12,13]
Handling	Food handler	Source	*Staphylococcus aureus*, Noroviruses, hepatitis A, *E. coli*, Shiga toxin-producing *E. coli* (STEC), *Salmonella* sp.	[45,46]

**Table 2:** Pathogens identified in different types of sandwiches.

Sandwich Type	Pathogen	References
Cheeseburger	*Listeria monocytogenes*	[60]
Hotdog sausages	*Salmonella* sp.	[60]
Chicken and meat shawarma	*L. monocytogenes*	[58]
Liver and minced meat	*Salmonella Enteritidis*, *S. Typhimurium*, *S. Dublin*, *Shigella flexneri* and *S. dysenteriae*	[61]
Seafood	*Staphylococcus aureus*, *Bacillus cereus*	[62]
Hotdog	*E. coli* and *B. cereus*	[63]
Chicken	Methicillin-resistant *S. aureus*	[68]
Deli slices	*L. monocytogenes*	[69]
Shawarma	*S. aureus*	[70]
Meat shawarma	*S. aureus*	[71]
Meat	*Escherichia coli*, *Klebsiella pneumoniae*	[72]
Eggs	*E. coli*, *K. pneumoniae*	[72]

**Table 3:** Microbial hazards, growth potential, and risk factors in sandwiches prepared under cook–serve and cook–chill systems.

System	Examples of Microbial Hazards	Growth Potential	Risk Factors	References
Cook–serve	*Escherichia coli*	Generally low if proper cooking and handling are maintained	Cross-contamination from food contact surfaces and chefs’ hands.	[73]
	*Campylobacter*	High risk if hygiene is not maintained	Non-compliance with hygiene standards on food contact surfaces and chefs’ hands.	[67]
	*Total coliforms*	Can grow if temperature control is inadequate	Poor hand hygiene and improper sanitation practices.	[67]
Cook–chill	*Clostridium perfringens*	High if cooling is not rapid and effective	Inadequate cooling processes.	[74]
	*Listeria monocytogenes*	Can grow at refrigeration temperatures	Extended storage duration and temperature abuse.	[75,76,77]
	*Bacillus cereus*	Can grow if temperature control is inadequate	Improper holding temperature and cross-contamination.	[69]
	*Staphylococcus aureus*	Can grow if post-cooking handling is poor	Addition of ingredients after cooking and poor sanitation.	[78,79]
	*Salmonella*	Low if proper cooking and handling are maintained	Cross-contamination and improper sanitation.	[78]
	*E. coli*	Can grow if temperature control is inadequate	Extended storage duration and inadequate chilling.	[20]

**Table 4:** Common virulence genes in sandwich-isolated pathogens.

Pathogen	Virulence Gene	References
*Bacillus cereus*	*Nhe*, *hbl*, *CytK*	[97]
*Staphylococcus aureus*	*sea* to *see* and *seh*	[96,105,106,82]
*Listeria monocytogenes*	*InlA*, *prfA*, *hly*	[103,104]
*E. coli* (ETEC)	*sth*, *stp*, *lt*	[98,99]
*E. coli* (EPEC)	*eaeA*, *bfpA*	[98,99]
*E. coli* (EAEC)	*aggR*	[98,99]
*E. coli* (EIEC)	*ipaH*	[98,99]
*E. coli* (DAEC)	*daaE*	[98,99]
*E. coli* (STEC)	*stx1a*, *stx1c*, *stx1d*, *stx2a*, *stx2b*, *stx2c*, *stx2d*, *stx2e*, *stx2f*, *stx2g*	[93,17,98,100,108]

**Table 5:** Antibiotic resistance in various foodborne pathogens recovered from sandwiches.

Sandwich Type	Location	Bacteria	Antibiotic Resistance	References
Shawarma (chicken with tahini sauce and vegetables)	Klang Valley, Malaysia	*Staphylococcus aureus*	Ampicillin, penicillin, ciprofloxacin, tetracycline, kanamycin, trimethoprim, trimethoprim–sulfamethoxazole, gentamicin, cephalothin	[70]
Kofta, luncheon, burger, shawarma, hawawshi, liver, sausage	Zagazig, Egypt	*S. aureus*	Kanamycin, penicillin, neomycin, oxacillin, erythromycin, ampicillin, nalidixic acid	[72]
Meat, chicken, and fish	Sharkia Governorate, Egypt	*Escherichia coli*	Erythromycin, amoxicillin–clavulanic acid	[121]
Meat (kebab, hamburger)	Iran	*E. coli*, *Salmonella* spp., *S. aureus*, *Listeria monocytogenes*	Amoxicillin, penicillin, cefalexin	[124]
Shawarma (chicken and beef)	Jordan	*E. coli*, *Salmonella* spp., *Citrobacter freundii*, *S. aureus*	Tetracycline, streptomycin	[125]
Various (tuna and tomato, ham and cheese, tomato and mozzarella cheese, tuna and eggs, turkey and vegetables, shrimp and pink sauce, raw ham, smoked cheese and tomatoes, cooked ham and mushrooms, tuna and onions, cooked ham and artichokes, raw ham and eggplants)	Modena, Italy	*S. aureus*, *Listeria* spp. *Yersinia* spp. *Citrobacter* spp.	Amikacin, ciprofloxacin, ampicillin, oxacillin, imipenem, tetracycline, erythromycin, vancomycin	[11]

## Data Availability

The original contributions presented in this study are included in the article. Further inquiries can be directed to the corresponding author.

## Abbreviations

The following abbreviations are used in this manuscript:

RTE	Ready-to-eat food
EHEC	Enterohemorrhagic *E. coli*
ETEC	Enterotoxigenic *E. coli*
EAEC	Enteroaggregative *E. coli*
EIEC	Enteroinvasive *E. coli*
DAEC	Diffuse-adherent *E. coli*
MIC	Minimum inhibitory concentration
ARB	Antibiotic-resistant bacteria
MRSA	Multidrug-resistant *S. aureus*
VRSA	Vancomycin-resistant *S. aureus*
MDR	Multidrug-resistant
ESBL	Extended-Spectrum β-Lactamases
AmpC	AmpC β-Lactamases
ARG	Antibiotic resistance genes
ATP	Adenosine triphosphate
PMF	Proton-motive force
MFS	Major facilitator superfamily
SMR	Small multidrug resistance family
HGT	Horizontal gene transfer

## References

[1] Codex Alimentarius Commission (1997). Regional Guidelines for the Design of Control Measures for Street-Vended Foods (Africa) CAC/GL 22R-1997. https://www.fao.org/4/w6419e/w6419e05.htm.

[2] Necidová L., Bursová Š., Haruštiaková D., Bogdanovičová K. (2022). Evaluation of *Staphylococcus aureus* growth and staphylococcal enterotoxin production in delicatessen and fine bakery products. Acta Vet. Brno.

[3] Huang J., Luo Y., Zhou B., Zheng J., Nou X. (2019). Growth and survival of *Salmonella enterica* and *Listeria monocytogenes* on fresh-cut produce and their juice extracts: Impacts and interactions of food matrices and temperature abuse conditions. Food Control.

[4] Min K.-J., Jung Y.-J., Kwon K.-Y., Kim J.-H., Hwang I.-G., Yoon K.-S. (2013). Effect of temperature on the production of staphylococcal enterotoxin and thermal inactivation kinetics of *Staphylococcus aureus* in selected ready-to-eat (RTE) foods in Korea. J. Food Saf..

[5] Onohuean H., Olot H., Onohuean F.E., Bukke S.P.N., Akinsuyi O.S., Kade A. (2025). A scoping review of the prevalence of antimicrobial-resistant pathogens and signatures in ready-to-eat street foods in Africa: Implications for public health. Front. Microbiol..

[6] Hwang C.A., Juneja V. (2011). Effects of salt, sodium pyrophosphate, and sodium lactate on the probability of growth of *Escherichia coli* O157:H7 in ground beef. J. Food Prot..

[7] Söderqvist K., Tauson H., Vågsholm I., Tysklind A., Wallenbeck A., Kaden R., Hultén K. (2017). Fate of *Listeria monocytogenes*, pathogenic *Yersinia enterocolitica*, and *Escherichia coli* O157:H7 gfp+ in ready-to-eat salad during cold storage: What is the risk to consumers?. J. Food Prot..

[8] Rodríguez-Caturla M.Y., Valero A., García-Gimeno R.M., Zurera G. (2012). Development of a risk-based methodology for estimating survival and growth of enteropathogenic *Escherichia coli* on iceberg-lettuce exposed at short-term storage in foodservice centers. J. Microbiol. Methods.

[9] Malak N.M.L., Soliman N.S.M. (2021). The effect of time and temperature variations on the microbial load and deterioration criteria of leftover cheeseburger sandwiches. Adv. Anim. Vet. Sci..

[10] Mahyuddin N.A., Nadia H., Wan-Zunairah W.I. (2014). Evaluation of hygiene practices on microbiological quality of tuna sandwiches. Theory and Practice in Hospitality and Tourism Research.

[11] Camellini S., Iseppi R., Condò C., Messi P. (2021). Ready-to-Eat Sandwiches as Source of Pathogens Endowed with Antibiotic Resistance and Other Virulence Factors. Appl. Sci..

[12] Shah S.P., Wu M.C., Ho T.T., Chen J.K., Chow M.H., Ko W.C., Hu S.W., Huang Y.F., Chiu C.S. (2020). Insights into the Microbiological Safety of Wooden Cutting Boards Used for Meat Processing in Hong Kong’s Wet Markets: A Focus on Food-Contact Surfaces, Cross-Contamination and the Efficacy of Traditional Hygiene Practices. Microorganisms.

[13] Al-Mutairi A.N., Enebe P.U., Al-Fouzan M.A., Eed F.A. (2016). The Prevalence of Foodborne Pathogenic Bacteria on Cutting Boards and Their Ecological Correlation with Background Biota. AIMS Microbiol..

[14] Eng S.K., Pusparajah P., Ab Mutalib N.S., Ser H.L., Chan K.G., Lee L.H. (2015). *Salmonella*: A Review on Pathogenesis, Epidemiology and Antibiotic Resistance. Front. Life Sci..

[15] Al-Kharousi Z.S., Guizani N., Al-Sadi A.M., Al-Bulushi I.M., Shaharoona B. (2016). Hiding in Fresh Fruits and Vegetables: Opportunistic Pathogens May Cross Geographical Barriers. Int. J. Microbiol..

[16] Liu W., Ma K., Zhu C., Zhang M., Guo B., Yang M., Li B. (2021). Isolation, Identification and Diversity of Lactic Acid Bacteria from Fermented Olive Juice in Morocco. Sci. Technol. Food Ind..

[17] Wei W.S., Huang A.S., Liao Y.-S., Liu Y.-N., Chiou C.-S. (2014). A large outbreak of salmonellosis associated with sandwiches contaminated with multiple bacterial pathogens purchased via an online shopping service. Foodborne Pathog. Dis..

[18] Davis S., King M.G., Casson J.W., Gray J.O., Caldwell D.G. (2007). End effector development for automated sandwich assembly. Meas. Control.

[19] Abidi S.M., Yamani M.I. (2024). Microbiological and chemical profiles of retail falafel sandwich in Jordan. Afr. J. Food Agric. Nutr. Dev..

[20] Kokkinakis M.N., Fragkiadakis G.A., Lapidakis N.E., Kokkinaki A.N. (2020). Assessing microbiological quality of ready-to-eat prepacked sandwiches, in Crete, Greece. J. Food Sci. Technol..

[21] Kumar A. (2015). Polyphasic characterization of microbial community from the surface of *Brassica oleracea* (Cabbage) head. J. Pure Appl. Microbiol..

[22] Pérez-Cataluña A., Liserra T., De la Haba A.F., Moreno-Indias I., Cardenas-Loeza B.E., Arévalo-Villena M., García-Ruiz A., García-Martínez T. (2018). Diversity and dynamics of lactic acid bacteria in *Atole agrio*, a traditional maize-based fermented beverage from South-Eastern Mexico, analysed by high throughput sequencing and culturing. Antonie Leeuwenhoek.

[23] Genaro A., Juliana M., Savilo J. (2020). Isolation and identification of thermophilic lactobacilli from various species of dairy. Acta Microsc..

[24] Cantoni C., Stella S., Cozzi M., Iacumin L., Comi G. (2004). Bacterial flora isolated on surfaces of ‘ricotta’ and of mould and smear ripened cheeses. Ind. Aliment..

[25] Cantoni C., Stella S., Iacumin L., Comi G. (2003). Red coloured cheeses and related bacteria. Ind. Aliment..

[26] Gargano V., Scuotto M., Romano A., Aponte M., Cocolin L., Blaiotta G., D’Auria M.V., Succi M., Tremonte P. (2024). Tracking the transfer of antimicrobial resistance genes from raw materials to sourdough breads. Food Biosci..

[27] Zeng X., Wang T., Li X., Li Q., Zhu X. (2023). Effects of different salt concentrations on microbial community in sauce mash and quality of soy sauce. China Brew..

[28] Devanthi P.V.P., Gkatzionis K. (2019). Soy sauce fermentation: Microorganisms, aroma formation, and process modification. Food Res. Int..

[29] Arslan S., Eyi A., Özdemir F. (2011). Spoilage potentials and antimicrobial resistance of *Pseudomonas* spp. isolated from cheeses. J. Dairy Sci..

[30] Chen B., Mei J., Xie J. (2024). Research on the spoilage potential and metabolic patterns of specific spoilage bacteria in grouper (*Epinephelus lanceolatus*) during cold storage. Food Biosci..

[31] Fang J., Feng L., Lu H., Zhu J. (2022). Metabolomics reveals spoilage characteristics and interaction of *Pseudomonas lundensis* and *Brochothrix thermosphacta* in refrigerated beef. Food Res. Int..

[32] Zhou Z., Li X., Zhu T., Lu F., Zhang J., Wang J., Wu Z., Niu C. (2024). Characterization and interactions of spoilage of *Pseudomonas fragi* C6 and *Brochothrix thermosphacta* S5 in chilled pork based on LC-MS/MS and screening of potential spoilage biomarkers. Food Chem..

[33] Sini K., Skandamis P., Nychas G. (2010). Shelf-life establishment of dairy products based on quality and safety determinants. J. Environ. Prot. Ecol..

[34] Pothakos V., Björkroth J., Lahtinen S., Ouwehand A.C., Salminen S., Von Wright A. (2019). Lactic Acid Bacteria in Food Spoilage. Lactic Acid Bacteria: Microbiological and Functional Aspects.

[35] Cenci-Goga B.T., Manuzza M.E., Donato F., Ciliberti A., Balzaretti E., Varisco G., Ranucci S. (2020). Characterization and growth under different storage temperatures of ropy slime-producing *Leuconostoc mesenteroides* isolated from cooked meat products. J. Food Prot..

[36] Snyder A.B., Biango-Daniels M.N., Hodge K.T., Worobo R.W. (2019). Nature Abhors a Vacuum: Highly Diverse Mechanisms Enable Spoilage Fungi to Disperse, Survive, and Propagate in Commercially Processed and Preserved Foods. Compr. Rev. Food Sci. Food Saf..

[37] Pellegrini M., Morello R., Roccato A., Iacumin L., Cozzi M., Zapparoli G., Manzocco L., Comi G. (2025). Chalk Yeasts Cause Gluten-Free Bread Spoilage. Microorganisms.

[38] Silva S., Rocha S., Rocha R. (2023). *Salmonella* infection in poultry: A review on the pathogen and control strategies. Microorganisms.

[39] Uzal F.A., Henderson E., Asin J., Alcaide J. (2024). Botulism in fish: A review. J. Vet. Diagn. Investig..

[40] Trudel-Ferland M., Levasseur M., Goulet-Beaulieu V., Jubinville E., Hamon F., Jean J. (2024). Concentration of foodborne viruses eluted from fresh and frozen produce: Applicability of ultrafiltration. Int. J. Food Microbiol..

[41] Seymour I.J., Appleton H. (2001). Foodborne viruses and fresh produce. J. Appl. Microbiol..

[42] Jääskeläinen E.L., Häggblom M.M., Andersson M.A., Vanne L., Salkinoja-Salonen M.S. (2003). Potential of *Bacillus cereus* for producing an emetic toxin, cereulide, in bakery products: Quantitative analysis by chemical and biological methods. J. Food Prot..

[43] Shivanandappa M., Harisha H., Veena K. (2023). Investigation of food poisoning outbreak in a temporary camp using mixed methodology approach. Asian J. Pharm. Clin. Res..

[44] Little C.L., Omotoye R., Mitchell R.T. (2003). The microbiological quality of ready-to-eat foods with added spices. Int. J. Environ. Health Res..

[45] Pexara A., Govaris A. (2020). Foodborne Viruses and Innovative Non-Thermal Food-Processing Technologies. Foods.

[46] Sollid J.U., Furberg A.S., Hanssen A.M., Johannessen M. (2014). *Staphylococcus aureus*: Determinants of human carriage. Infect. Genet. Evol..

[47] Jonge R. (2019). Predictable and unpredictable survival of foodborne pathogens during non-isothermal heating. Int. J. Food Microbiol..

[48] Bejerholm C., Tørngren M.A., Aaslyng M.D., Dikeman M.E., Devine C. (2014). Cooking of meat. Encyclopedia of Meat Sciences.

[49] Djimsa B.A., Abraham A., Mafi G.G., VanOverbeke D.L., Ramanathan R. (2017). Effects of metmyoglobin reducing activity and thermal stability of NADH-dependent reductase and lactate dehydrogenase on premature browning in ground beef. J. Food Sci..

[50] Sood S., Sharma C. (2019). Bacteria in Indian food packaging papers and paperboards with various contents of pulp fiber. Food Nutr. Sci..

[51] Mengistu D.A., Belami D.D., Tefera A.A., Asefa Y.A. (2022). Bacteriological Quality and Public Health Risk of Ready-to-Eat Foods in Developing Countries: Systematic Review and Meta Analysis. Microbiol. Insights.

[52] Büyükyörük S., Beyaz D., Göksoy E.Ö., Kök F., Koçak P. (2014). Microbiological evaluation of ready-to-eat sandwiches served near hospitals and schools. Ank. Üniv. Vet. Fak. Derg..

[53] Borrusso P.A., Quinlan J.J. (2017). Prevalence of Pathogens and Indicator Organisms in Home Kitchens and Correlation with Unsafe Food Handling Practices and Conditions. J. Food Prot..

[54] Bell R.L., Dreesman E., Gräbel F., Hoelzer K. (2021). The Persistence of Bacterial Pathogens in Surface Water and Its Impact on Global Food Safety. Pathogens.

[55] Sosah F.K., Odoom A., Anim-Baidoo I., Donkor E.S. (2025). How long do pathogens persist and survive in water? A systematic review. Front. Microbiol..

[56] Zormati S., Kallel I., Sellami H., Gdoura R. (2018). Influence of environmental and seasonal factors on microbial contamination levels in clam production areas in southern Tunisia: *Escherichia coli*, *Salmonella* spp., hepatitis A virus and norovirus. Rev. Sci. Tech..

[57] Aghalari Z., Hosseini S.R., Jafarian S., Rezazadeh M., Mirzaei M., Esmaeili E., Hasanzadeh P. (2021). Evaluation of chemical and microbial quality of food in northern Iran. J. Food Prot..

[58] Mohamed K.A.S., Abd-Allah S.M.S., Ismail H.A.M.A. (2022). Evaluation of hygienic and nutritional quality of kofta and sausage sandwiches in New Valley governorate. Assiut Vet. Med. J..

[59] Rhee S.J., Lee J.E., Lee C.H. (2011). Importance of lactic acid bacteria in Asian fermented foods. Microb. Cell Fact..

[60] Asiegbu C.V., Lebelo S.L., Tabit F.T. (2020). Microbial quality of ready-to-eat street vended food groups sold in the Johannesburg Metropolis, South Africa. J. Food Qual. Hazards Control.

[61] El-Shenawy M., El-Shenawy M., Mañes J., Soriano J.M. (2011). *Listeria* spp. in street-vended ready-to-eat foods. Interdiscip. Perspect. Infect. Dis..

[62] El-Rahman S.S., Samaha I.A., Haggag Y.N., Nossair M.A. (2018). Incidence of some pathogenic bacteria in fast food sandwiches. Alex. J. Vet. Sci..

[63] Fang T.J., Wei Q.K., Liao C.W., Hung M.J., Wang T.H. (2003). Microbiological quality of 18 °C ready-to-eat food products sold in Taiwan. Int. J. Food Microbiol..

[64] Hanashiro A., Morita M., Matté G.R., Matté M.H., Torres E.A. (2005). Microbiological quality of selected street foods from a restricted area of Sao Paulo city, Brazil. Food Control.

[65] Abd-El-Malek A. (2014). Microbiological quality of ready-to-eat liver sandwiches (Kibda). Glob. Vet..

[66] Meldrum R.J., Smith R.M., Ellis P., Garside J. (2006). Microbiological quality of randomly selected ready-to-eat foods sampled between 2003 and 2005 in Wales, UK. Int. J. Food Microbiol..

[67] Sprenger R. (2023). Hygiene for Management.

[68] Al-Rahbi A., Al-Bulushi I., Al-Rizeiki M., Al-Subhi L. (2025). Microbial characteristics of cook-chill sandwiches: Technical note. J. Agric. Mar. Sci..

[69] Elshebrawy H.A., Kasem N.G., Sallam K.I. (2025). Methicillin- and vancomycin-resistant *Staphylococcus aureus* in chicken carcasses, ready-to-eat chicken meat sandwiches, and buffalo milk. Int. J. Food Microbiol..

[70] Van Paepeghem C., Taghlaoui F., De Loy-Hendrickx A., Vermeulen A., Devlieghere F., Jacxsens L., Uyttendaele M. (2024). Prevalence and growth potential of *Listeria monocytogenes* in innovative, pre-packed, plant-based ready-to-eat food products on the Belgian market. Int. J. Food Microbiol..

[71] Fathalla M.S., Mahyudin N.A., Ghazali F.M., Rukayadi Y., Jaafar S.N. (2020). Detection of enterotoxin gene (*Sea*) and biofilm formation ability among multi-drug resistant *Staphylococcus aureus* isolated from shawarma sandwich sold at selected kiosks in Klang Valley, Malaysia. Food Res..

[72] Morshdy A.E.M.A., Hussein M.A., Tharwat A.E., Fakhry B.A. (2018). Prevalence of enterotoxigenic and multi-drug-resistant *Staphylococcus aureus* in ready to eat meat sandwiches. Slov. Vet. Res..

[73] Yaici L., Hacini M., Boukli N., Benchouiha N., Hadj-Aissa L., Hani K., Boukoum L. (2017). Spread of ESBL/AmpC-producing *Escherichia coli* and *Klebsiella pneumoniae* in the community through ready-to-eat sandwiches in Algeria. Int. J. Food Microbiol..

[74] Lai H., Lin G., Wang T., Zhang Y., Liu X., Huang J., Song S., Huang Y., Zhang R., Wang Z. (2024). Microbiological safety assessment of restaurants and HACCP-certified kitchens in hotels: A study in eastern China. Int. J. Food Microbiol..

[75] Poumeyrol G., Morelli E., Rosset P., Noel V. (2014). Probabilistic evaluation of *Clostridium perfringens* potential growth in order to validate a cooling process of cooked dishes in catering. Food Control.

[76] Tirloni E., Stella S., de Knegt L.V., Gandolfi G., Bernardi C., Nauta M.J. (2018). A quantitative microbial risk assessment model for *Listeria monocytogenes* in RTE sandwiches. Microb. Risk Anal..

[77] Georgalis L., Garre A., Fernandez Escamez P.S. (2020). Training in tools to develop Quantitative Risk Assessment using Spanish ready-to-eat food examples. EFSA J..

[78] Rosset P., Cornu M., Noël V., Morelli E., Poumeyrol G. (2004). Time-temperature profiles of chilled ready-to-eat foods in school catering and probabilistic analysis of *Listeria monocytogenes* growth. Int. J. Food Microbiol..

[79] Jang H.G., Kim N.H., Choi Y.M., Rhee M.S. (2013). Microbiological quality and risk factors related to sandwiches served in bakeries, Cafés, and sandwich bars in South Korea. J. Food Prot..

[80] Thomas J., Govender N., McCarthy K., Erasmus L., Doyle T., Allam M., Ismail A., Ramalwa N., Sekwadi P., Ntshoe G. (2020). Outbreak of listeriosis in South Africa associated with processed meat. N. Engl. J. Med..

[81] Al-Abri S.S., Al-Jardani A.K., Al-Hosni M.S., Kurup P.J., Al-Busaidi S., Beeching N.J. (2011). A hospital acquired outbreak of *Bacillus cereus* gastroenteritis in Oman. J. Infect. Public Health.

[82] Ercoli L., Gallina S., Nia Y., Auvray F., Primavilla S., Guidi F., Pierucci B., Graziotti C., Decastelli L., Scuota S. (2017). Investigation of a Staphylococcal Food Poisoning Outbreak from a Chantilly Cream Dessert, in Umbria (Italy). Foodborne Pathog. Dis..

[83] World Health Organization (2022). Multi-Country Outbreak of *Salmonella Typhimurium* Linked to Chocolate Products—Europe and the United States of America. https://www.who.int/emergencies/disease-outbreak-news/item/2022-DON369.

[84] Quinn O., Lane C., Hughes H.E., De Pinna E., Dallman T., Boxall E., Gobbi E., Charlett A., Insalata V., Chalmers R. (2024). National outbreak of Shiga toxin-producing *Escherichia coli* O145:H28 associated with pre-packed sandwiches, United Kingdom, May–June 2024. Epidemiol. Infect..

[85] Butt S., Lane C., Boxall E., Hughes H.E., Dallman T., Whitehead M., Gobbi E., Charlett A., Chalmers R., Insalata V. (2021). Epidemiological investigations identified an outbreak of Shiga toxin-producing *Escherichia coli* serotype O26:H11 associated with pre-packed sandwiches. Epidemiol. Infect..

[86] Kashima K., Sato M., Osaka Y., Sakakida N., Kando S., Ohtsuka K., Doi R., Chiba Y., Takase S., Fujiwara A. (2021). An outbreak of food poisoning due to *Escherichia coli* serotype O7:H4 carrying *astA* for enteroaggregative *E. coli* heat-stable enterotoxin 1 (EAST1). Epidemiol. Infect..

[87] Ortega-Benito J.M., Langridge P. (1992). Outbreak of food poisoning due to *Salmonella typhimurium* DT4 in mayonnaise. Public Health.

[88] Whitworth J. Hong Kong Outbreak Linked to Sandwiches Sickens 200 *Food Safety News*. 25 October 2023. https://www.foodsafetynews.com/2023/10/hong-kong-outbreak-linked-to-sandwiches-sickens-200/.

[89] Food and Drug Administration (2025). Outbreak Investigation of *Listeria monocytogenes*: Ready-to-Eat Foods. https://www.fda.gov/food/outbreaks-foodborne-illness/outbreak-investigation-listeria-monocytogenes-ready-eat-foods-may-2025.

[90] Food Safety News Spanish Food Poisoning Cases Linked to Sandwich; One Person Dead *Food Safety News*. 10 October 2024. https://www.foodsafetynews.com/2024/10/spanish-food-poisoning-cases-linked-to-sandwich-one-person-dead/.

[91] Little C.L., Amar C.F., Awofisayo A., Grant K.A. (2012). Hospital-acquired listeriosis associated with sandwiches in the UK: A cause for concern. J. Hosp. Infect..

[92] Hedican E., Hoekstra R.M., Kretzschmar K., Ehlers J., Smith S.B., Knipe B., Jones T.F. (2009). Restaurant *Salmonella enteritidis* outbreak associated with an asymptomatic infected food worker. J. Food Prot..

[93] Newell K., Helfrich K., Isernhagen H., Jones M., Stickel G., McKeel H., Castrodale L., McLaughlin J. (2024). Multipathogen Outbreak of *Bacillus cereus* and *Clostridium perfringens* Among Hospital Workers in Alaska, August 2021. Public Health Rep..

[94] Khater-Dalia F., Heikal G.E., Shehata A.A., El-Hofy F.I. (2014). The microbiological assessment of ready-to-eat food (liver and kofta sandwiches) in Tanta City. Benha Vet. Med. J..

[95] (2015). Microbiological Criteria for Foodstuffs.

[96] Alberta Health Services (AHS) (2011). Microbial Guidelines for Ready-to-Foods.

[97] Zhang Z., Feng L., Xu H., Liu C., Shah N., Wei H. (2016). Detection of viable enterotoxin-producing *Bacillus cereus* and analysis of toxigenicity from ready-to-eat foods and infant formula milk powder by multiplex PCR. J. Dairy Sci..

[98] Oh K., Kim S., Park M., Cho S. (2014). Development of a one-step PCR assay with nine primer pairs for the detection of five diarrheagenic *Escherichia coli* types. J. Microbiol. Biotechnol..

[99] Vidal M., Kruger E., Durán C., Lagos R., Levine M., Prado V., Toro C., Vidal R. (2005). Single multiplex PCR assay to identify simultaneously the six categories of diarrheagenic *Escherichia coli* associated with enteric infections. J. Clin. Microbiol..

[100] Food Safety Authority of Ireland Scientific Committee (2019). Advice on Shiga Toxin-Producing Escherichia coli (STEC) Detection in Food.

[101] Food and Agriculture Organization of the United Nations, World Health Organization (2018). Shiga Toxin-Producing Escherichia coli (STEC) and Food: Attribution, Characterization and Monitoring.

[102] Smith J., Fratamico P.M., Trugo L., Finglas P. (2016). *Escherichia coli* and Other Enterobacteriaceae: Food Poisoning and Health Effects. Encyclopedia of Food and Health.

[103] Poimenidou S., Dalmasso M., Papadimitriou K., Fox E., Skandamis P., Jordan K. (2018). Virulence gene sequencing highlights similarities and differences in sequences in *Listeria monocytogenes* serotype 1/2a and 4b strains of clinical and food origin from 3 different geographic locations. Front. Microbiol..

[104] Peiris W. (2005). *Listeria monocytogenes*, a Food-Borne Pathogen. Master’s Thesis.

[105] Haghi F., Zeighami H., Hajiloo Z., Derakhshan S. (2021). High frequency of enterotoxin encoding genes of *Staphylococcus aureus* isolated from food and clinical samples. J. Health Popul. Nutr..

[106] Le H.H.T., Dalsgaard A., Andersen P.S., Nguyen H.M., Ta Y.T., Nguyen T.T. (2021). Large-scale *Staphylococcus aureus* foodborne disease poisoning outbreak among primary school children. Microbiol. Res..

[107] Ikeda T., Omoe F.I., Nakano M.S., Omoe H.B., Sekiguchi N.K. (2005). Characterization of *Staphylococcus aureus* strains and evidence for the involvement of non-classical enterotoxin genes in food poisoning outbreaks. FEMS Microbiol. Lett..

[108] Alharbi S.A., Abdel-Ghaffar M.H., Kadher N.R. (2019). Isolation and identification of pathogenic bacteria from ready-to-eat fast foods in Al-Quwayiyah, Kingdom of Saudi Arabia. Afr. J. Food Agric. Nutr. Dev..

[109] Hernández M., Borrull F., Calull M. (2003). Analysis of antibiotics in biological samples by capillary electrophoresis. TrAC Trends Anal. Chem..

[110] Teuber M. (1999). Spread of antibiotic resistance with food-borne pathogens. Cell. Mol. Life Sci..

[111] Brauner A., Fridman O., Gefen O., Balaban N.Q. (2016). Distinguishing between resistance, tolerance and persistence to antibiotic treatment. Nat. Rev. Microbiol..

[112] Kawane R.S. (2012). Studies on antibiotics and heavy metal resistance profiling of *Escherichia coli* from drinking water and clinical specimens. Biosci. Discov..

[113] Magnano San Lio R., Favara G., Maugeri A., Barchitta M., Agodi A. (2023). How antimicrobial resistance is linked to climate change: An overview of two intertwined global challenges. Int. J. Environ. Res. Public Health.

[114] Burnham J.P. (2021). Climate change and antibiotic resistance: A deadly combination. Ther. Adv. Infect. Dis..

[115] Kaba H.E.J., Kuhlmann E., Scheithauer S. (2020). Thinking outside the box: Association of antimicrobial resistance with climate warming in Europe—A 30 country observational study. Int. J. Hyg. Environ. Health.

[116] Al-Bayati Z.A.K., El-Sayed A.I. (2023). Compounding Effects of Climate Change and Antibiotic Resistance. Iraq J. Sci..

[117] Deng Y., Xu H., Su Y., Liu S., Xu L., Guo Z., Wu J., Cheng C., Feng J. (2019). Horizontal gene transfer contributes to virulence and antibiotic resistance of *Vibrio harveyi* 345 based on complete genome sequence analysis. BMC Genom..

[118] Kayode A.J., Okoh A.I. (2023). Antimicrobial-resistant *Listeria monocytogenes* in ready-to-eat foods: Implications for food safety and risk assessment. Foods.

[119] Salimi Y., Mirzavand B., Ayoubi J. (2014). Determination of bacterial contamination isolated from sandwiches in Kerman City and their resistance to commonly used antimicrobials. Arch. Appl. Sci. Res..

[120] Ajaja S.A., Bozakoukb I.H., Abdalla I.I., Bumadian M.M., Bleiblo A.M., Keand I.R. (2020). Bacterial contamination of fast food shawarma sandwiches in local restaurants in Benghazi city, Libya. Libyan J. Sci. Technol..

[121] Hussein M., Eldaly E., Seadawy H., El-Nagar E. (2018). Virulence and antimicrobial resistance genes of *Escherichia coli* in ready to eat sandwiches in Sharkia governorate. Slov. Vet. Res..

[122] Khan M.F., Al-Shihi A.A., Al-Zaabi A.S., Bakheit Z. (2023). Antimicrobial stewardship opportunities: An example from Oman. Oman Med. J..

[123] Mesbah A., Mashak Z., Abdolmaleki Z. (2021). A survey of prevalence and phenotypic and genotypic assessment of antibiotic resistance in *Staphylococcus aureus* bacteria isolated from ready-to-eat food samples collected from Tehran Province, Iran. Trop. Med. Health.

[124] Rajaei M., Moosavy M.H., Gharajalar S.N., Khatibi S.A. (2021). Antibiotic resistance in the pathogenic foodborne bacteria isolated from raw kebab and hamburger: Phenotypic and genotypic study. BMC Microbiol..

[125] Nimri L., Dahab F.A.A., Batchoun R. (2014). Foodborne bacterial pathogens recovered from contaminated shawarma meat in northern Jordan. J. Infect. Dev. Ctries..

[126] Van Schaik W. (2015). The human gut resistome. Philos. Trans. R. Soc. B Biol. Sci..

[127] Chattopadhyay M.K. (2011). Antibiotic and heavy metal resistance of bacterial isolates obtained from some lakes in northern Germany. NSHM J. Pharm. Healthc. Manag..

[128] Krishna M.P., Varghese R., Hatha A.A.M. (2016). Heavy metal tolerance and multiple drug resistance of heterotrophic bacterial isolates from metal contaminated soil. S. Pac. J. Nat. Appl. Sci..

[129] Martínez J.L. (2008). Antibiotics and antibiotic resistance genes in the environment: An ecosystem perspective. Environ. Pollut..

[130] Blair J.M.A., Webber M.A., Baylay A.J., Ogbolu D.O., Piddock L.J.V. (2015). Molecular mechanisms of antibiotic resistance. Nat. Rev. Microbiol..

[131] Cox G., Wright G.D. (2013). Intrinsic antibiotic resistance: Mechanisms, origins, challenges and solutions. Int. J. Med. Microbiol..

[132] Horner C., Mawer D., Wilcox M. (2012). Reduced susceptibility to chlorhexidine in staphylococci: Is it increasing and does it matter?. J. Antimicrob. Chemother..

[133] Gang Z., Jie F. (2016). The intrinsic resistance of bacteria. Yi Chuan.

[134] Zhang G., Ma Y., Zhang R., Xu X., Wang M., Lv J., Ma L. (2017). A new subclass of intrinsic aminoglycoside nucleotidyltransferases, ANT(3″)-II, is horizontally transferred among *Acinetobacter* spp. by homologous recombination. PLoS Genet..

[135] Woodford N., Ellington M.J. (2007). The emergence of antibiotic resistance by mutation. Clin. Microbiol. Infect..

[136] Roe M.T., Pillai S.D. (2003). Monitoring and identifying antibiotic resistance mechanisms in bacteria. Poult. Sci..

[137] Livermore D.M., Brown D.F.J. (2001). Detection of beta-lactamase-mediated resistance. J. Antimicrob. Chemother..

[138] Kaboudari A., Aliakbarlu J., Mehdizadeh T. (2024). Simultaneous effects of food-related stresses on the antibiotic resistance of foodborne *Salmonella* serotypes. J. Food Prot..

[139] Colavecchio A., Cadieux B., Lo A., Goodridge L.D. (2017). Bacteriophages contribute to the spread of antibiotic resistance genes among foodborne pathogens of the Enterobacteriaceae family—A review. Front. Microbiol..

[140] Yang Y., Zhou L., Zhou B., Zhao Y. (2013). The horizontal transfer of genetic elements of antibiotic resistance in *Streptococcus thermophilus*. J. Pure Appl. Microbiol..

[141] Sukumar S., Roberts A.P., Martin F.E., Adler C.J. (2016). Metagenomic insights into transferable antibiotic resistance in oral bacteria. J. Dent. Res..

[142] Zhao M., Gu X., Chen S., Gu H. (2025). Identification of outer membrane vesicles as a new vehicle mediating antibiotic resistance gene transfer in *Campylobacter*. J. Extracell. Vesicles.

[143] Chopra I., Roberts M. (2001). Tetracycline antibiotics: Mode of action, applications, molecular biology, and epidemiology of bacterial resistance. Microbiol. Mol. Biol. Rev..

[144] Johnsen P.J., Townsend J.P., Bøhn T., Simonsen G.S., Sundsfjord A., Nielsen K.M. (2011). Retrospective evidence for a biological cost of vancomycin resistance determinants in the absence of glycopeptide selective pressures. J. Antimicrob. Chemother..

[145] Gasparrini A.J., Ciorba M.A., Giles C., Ridenhour C.A., Dantas G. (2020). Tetracycline-inactivating enzymes from environmental, human commensal, and pathogenic bacteria cause broad-spectrum tetracycline resistance. Commun. Biol..

[146] Li X., Wang H.H. (2010). Tetracycline resistance associated with commensal bacteria from representative ready-to-consume deli and restaurant foods. J. Food Prot..

[147] Jung Y., Matthews K. (2016). Potential transfer of extended spectrum β-lactamase encoding gene, blashv18gene, between *Klebsiella pneumoniae* in raw foods. Food Microbiol..

[148] Shousha A., Ibrahem R., El-Baradei A., El-Said S., Awad A. (2015). Bacteriophages isolated from chicken meat and the horizontal transfer of antimicrobial resistance genes. Appl. Environ. Microbiol..

[149] Paula A.C.L., Medeiros J.D., Azevedo A.C., De Assis Chagas J.M., Silva V.L., Diniz C.G. (2018). Antibiotic resistance genetic markers and integrons in white soft cheese: Aspects of clinical resistome and potentiality of horizontal gene transfer. Genes.

[150] Wang H., Liu J., Jiang Y., Xu F., He F., Jiang J., Fang B. (2025). Metagenomics and binning analysis unveiled the diversity and transfer patterns of antibiotic resistance genes in traditional pickled vegetables. Food Biosci..

[151] Kumarasamy K.K., Toleman M.A., Walsh T.R., Bagaria J., Butt F., Balakrishnan R., Chaudhry U., Doumith P., Giske C.G., Irfan S. (2010). Emergence of a new antibiotic resistance mechanism in India, Pakistan, and the UK: A molecular, biological, and epidemiological study. Lancet Infect. Dis..

[152] Hölzel C.S., Tetens J.L., Schwaiger K. (2018). Unraveling the role of vegetables in spreading antimicrobial-resistant bacteria: A need for quantitative risk assessment. Foodborne Pathog. Dis..

[153] Forslund K., Sunagawa S., Bork P. (2013). Country-specific antibiotic use practices impact the human gut resistome. Genome Res..

[154] Karami N., Nowrouzian F., Adlerberth I., Wold A.E. (2006). Tetracycline resistance in *Escherichia coli* and persistence in the infantile colonic microbiota. Antimicrob. Agents Chemother..

[155] Zhang R., Eggleston K., Rotimi V., Zeckhauser R.J. (2006). Antibiotic resistance as a global threat: Evidence from China, Kuwait and the United States. Glob. Health.

[156] Chavan P., Vashishth R. (2025). Antimicrobial resistance in foodborne pathogens: Consequences for public health and future approaches. Discov. Appl. Sci..

[157] Negi S., Sharma S. (2024). Ready to Eat Food: A Reason for enhancement in multidrug resistance in humans. Adv. Pharm. Bull..

[158] Ntshanka Z., Ekundayo T.C., du Plessis E.M., Korsten L., Okoh A.I. (2022). Occurrence and molecular characterization of multidrug-resistant vegetable-borne *Listeria monocytogenes* isolates. Antibiotics.

[159] Durán G.M., Marshall D.L. (2005). Ready-to-eat shrimp as an international vehicle of antibiotic-resistant bacteria. J. Food Prot..

[160] Alsayeqh A.F. (2025). Antibiotic resistance and emerging alternatives for controlling foodborne pathogens. Pak. Vet. J..

[161] Acar J.F., Moulin G. (2013). Integrating animal health surveillance and food safety: The issue of antimicrobial resistance. OIE Rev. Sci. Tech..

[162] McEwen S.A., Aarestrup F.M., Jordan D. (2019). Monitoring of antimicrobial resistance in animals: Principles and practices. Antimicrobial Resistance in Bacteria of Animal Origin.

[163] Khan R.T., Bhardwaj M., Kailoo S., Khajuria R., Rasool S. (2024). A statistical study on awareness of antibiotic resistance among the general population. Indian J. Biochem. Biophys..

[164] Miłobędzka A., van Schaik W., Dutilh B.E., van der Oost J. (2022). Monitoring antibiotic resistance genes in wastewater environments: The challenges of filling a gap in the One-Health cycle. J. Hazard. Mater..

[165] Simões A.S., Candeias S., Bártolo I., Ramalheira E., Pinto C., Pinhão M.J., Almeida M.A., Pires M., Lameiras A., Simões H. (2018). Participatory implementation of an antibiotic stewardship programme supported by an innovative surveillance and clinical decision-support system. J. Hosp. Infect..

[166] Foxman B., Bair B.T., Soge O.O., Soneja S., Perin J., Karch J.M., Marra G.V., Geller B., Gentry H., Lye E.S. (2025). Wastewater surveillance of antibiotic-resistant bacteria for public health action: Potential and challenges. Am. J. Epidemiol..

[167] Zheng S., Li Y., Chen C., Wang N., Yang F. (2025). Solutions to the dilemma of antibiotics use in livestock and poultry farming: Regulation policy and alternatives. Toxics.

[168] Patel S.J., Wellington M., Shah R.M., Ferreira M.J. (2020). Antibiotic stewardship in food-producing animals: Challenges, progress, and opportunities. Clin. Ther..

[169] Marvasi M., Casillas L., Vassallo A., Purchase D. (2021). Educational activities for students and citizens supporting the one-health approach on antimicrobial resistance. Antibiotics.

[170] Kirchhelle C. (2018). Pharming animals: A global history of antibiotics in food production (1935–2017). Palgrave Commun..

[171] Lecky D.M., Hawking M.K.D., Verlander N.Q., McNulty C.A.M. (2014). Using interactive family science shows to improve public knowledge on antibiotic resistance: Does it work?. PLoS ONE.

[172] Bodie A.R., O’Bryan C.A., Olson E.G., Ricke S.C. (2023). Natural antimicrobials for *Listeria monocytogenes* in ready-to-eat meats: Current challenges and future prospects. Microorganisms.

[173] Silva F.V.M., Evelyn K. (2023). Pasteurization of food and beverages by high pressure processing (HPP) at room temperature: Inactivation of *Staphylococcus aureus*, *Escherichia coli*, *Listeria monocytogenes*, *Salmonella*, and other microbial pathogens. Appl. Sci..

[174] Popa E.E., Sărădan V.C., Pop I.M., Vlaic R.A., Mitel D.A., Sărădan M.B., Mureșan A.E. (2022). Antimicrobial active packaging containing nisin for preservation of products of animal origin: An Overview. Foods.

[175] Mina H.A., Buckley D.A., Burnett J., Deering A.J. (2025). Evaluation of commercially available produce antimicrobial washes to improve the quality and microbial safety of fresh produce. Int. J. Food Microbiol..

[176] Lippman B., Yao S., Huang R., Chen H. (2020). Evaluation of the combined treatment of ultraviolet light and peracetic acid as an alternative to chlorine washing for lettuce decontamination. Int. J. Food Microbiol..

[177] Saleh F.A., Al-Otaibi M.S., Alshammari N.F., Al-Habeeb A.A., Alturairi N., Albahrani S.H., Elshafey A. (2025). Comprehensive risk assessment and control measures in the food service chain of hospitals nutrition department: A case study in Al-Ahsa Governorate, Kingdom of Saudi Arabia. Front. Microbiol..

[178] Li T., Chen H., Zhao J., Tao Z., Lan W., Zhao Y., Sun X. (2023). Characterization of phage vB_SalM_SPJ41 and the reduction of risk of antibiotic-resistant *Salmonella enterica* contamination in two ready-to-eat foods. Antibiotics.

[179] Lima J.C.R., Silva L.H.F., Santos T.P., Leite E.R., Lima M.F., Salgado M.A.C., Santos I.M. (2023). X-ray computed microtomography time-dependent analysis of sandwich bread in different storage conditions. X-Ray Spectrom..

[180] Kothe C.I., Renault P. (2025). Metagenomic driven isolation of poorly culturable species in food. Food Microbiol..

